# An Imperfect Dopaminergic Error Signal Can Drive Temporal-Difference Learning

**DOI:** 10.1371/journal.pcbi.1001133

**Published:** 2011-05-12

**Authors:** Wiebke Potjans, Markus Diesmann, Abigail Morrison

**Affiliations:** 1 Institute of Neuroscience and Medicine (INM-6), Computational and Systems Neuroscience, Research Center Jülich, Jülich, Germany; 2 RIKEN Brain Science Institute, Wako-shi, Saitama, Japan; 3 Functional Neural Circuits Group, Faculty of Biology, Albert-Ludwig University of Freiburg, Freiburg, Germany; 4 Bernstein Center Freiburg, Albert-Ludwig University of Freiburg, Freiburg, Germany; 5 Brain and Neural Systems Team, RIKEN Computational Science Research Program, Wako-shi, Saitama, Japan; John Radcliffe Hospital, United Kingdom

## Abstract

An open problem in the field of computational neuroscience is how to link synaptic plasticity to system-level learning. A promising framework in this context is temporal-difference (TD) learning. Experimental evidence that supports the hypothesis that the mammalian brain performs temporal-difference learning includes the resemblance of the phasic activity of the midbrain dopaminergic neurons to the TD error and the discovery that cortico-striatal synaptic plasticity is modulated by dopamine. However, as the phasic dopaminergic signal does not reproduce all the properties of the theoretical TD error, it is unclear whether it is capable of driving behavior adaptation in complex tasks. Here, we present a spiking temporal-difference learning model based on the actor-critic architecture. The model dynamically generates a dopaminergic signal with realistic firing rates and exploits this signal to modulate the plasticity of synapses as a third factor. The predictions of our proposed plasticity dynamics are in good agreement with experimental results with respect to dopamine, pre- and post-synaptic activity. An analytical mapping from the parameters of our proposed plasticity dynamics to those of the classical discrete-time TD algorithm reveals that the biological constraints of the dopaminergic signal entail a modified TD algorithm with self-adapting learning parameters and an adapting offset. We show that the neuronal network is able to learn a task with sparse positive rewards as fast as the corresponding classical discrete-time TD algorithm. However, the performance of the neuronal network is impaired with respect to the traditional algorithm on a task with both positive and negative rewards and breaks down entirely on a task with purely negative rewards. Our model demonstrates that the asymmetry of a realistic dopaminergic signal enables TD learning when learning is driven by positive rewards but not when driven by negative rewards.

## Author Summary

What are the physiological changes that take place in the brain when we solve a problem or learn a new skill? It is commonly assumed that behavior adaptations are realized on the microscopic level by changes in synaptic efficacies. However, this is hard to verify experimentally due to the difficulties of identifying the relevant synapses and monitoring them over long periods during a behavioral task. To address this question computationally, we develop a spiking neuronal network model of actor-critic temporal-difference learning, a variant of reinforcement learning for which neural correlates have already been partially established. The network learns a complex task by means of an internally generated reward signal constrained by recent findings on the dopaminergic system. Our model combines top-down and bottom-up modelling approaches to bridge the gap between synaptic plasticity and system-level learning. It paves the way for further investigations of the dopaminergic system in reward learning in the healthy brain and in pathological conditions such as Parkinson's disease, and can be used as a module in functional models based on brain-scale circuitry.

## Introduction

Every higher organism needs to be able to make predictions about future rewards and adapt its behavior accordingly. One computational approach for modifying behavior to maximize reward on the basis of interactions with the environment is reinforcement learning [1]. Within that class of algorithms, temporal-difference (TD) learning, so called because it is based on comparing reward estimations at successive time steps, is particularly interesting to neuroscientists as it can solve tasks in which rewards or punishments are rare. Learning is driven by the TD error signal, which is positive when actions result in a condition that is better than expected, and negative if worse than expected.

Experimental findings, particularly on the dopaminergic system, support the hypothesis that the mammalian brain uses a TD learning strategy. During conditioning tasks, monkey midbrain dopamine neurons show phasic bursting activity following the presentation of an unpredicted reward. If, however, the reward is repeatedly paired with a reward predicting stimulus, the dopaminergic response shifts from the time of the reward delivery to the time of the stimulus onset. Furthermore, the dopaminergic activity decreases at the time of an expected reward if the reward is omitted [2], [3]. This phasic activity has strikingly similar characteristics to the TD error signal [2], [4], although other interpretations also exist [5]. Recently, dopamine-dependent prediction errors have also been observed in humans [6]. The main target for dopamine innervation is the striatum, the input area of the basal ganglia, where the released dopamine modulates the plasticity of synapses between the cortex and the striatum [7], [8]; see [9] for a review.

These results suggest that the basal ganglia play an important role in any implementation of TD learning in the brain. There is some evidence that the cortico-striatal circuit realizes a variant of TD learning known as the actor-critic architecture [10]. In this formulation of TD learning, explained in greater detail below, the agent learns an estimate for the amount of reward that can be gained starting from a given state [11], [12]. An alternative interpretation is that the agent learns the amount of reward that can be expected for a given choice of action [13], [14]. Regardless of the exact formulation of TD learning assumed, it is still unclear what the mechanisms are that would enable it to be implemented in the mammalian brain. Dopaminergic activity is typically recorded in classical conditioning [15], [16], instructed-choice instrumental conditioning [17] or simple decision trials with only a few number of possible actions [13]. In these tasks, a reward is delivered (sometimes delayed) after every (correct) action. Such experiments cannot tell us whether the phasic dopaminergic signal is able to guide learning in complex tasks with sparse reward.

This is a crucial point, as the phasic dopaminergic firing rate only resembles the error signal of TD learning to a limited extent. The most obvious difference between the two signals is that the low baseline firing rate of the dopamine neurons implies a lower bound for the representation of negative errors in the dopaminergic error signal, whereas the TD error is unbounded. To address the question of whether dopamine-dependent plasticity can implement TD learning on the basis of a dopaminergic signal, despite its deviations from a standard TD error, we use a computational model. In this way, we can study the dopaminergic error signal, the evolution of synapses subject to dopamine-dependent plasticity and the adaptation of behavior over a long time period in complex tasks. Previous models implementing TD learning by utilizing a dopaminergic signal have only been formulated for nonspiking neurons [4], [18]–[21] (for reviews see [22], [23]). Conversely, most existing spiking reinforcement learning models have focused on non-TD learning strategies [24]–[30]. Some of these non-TD models have been shown to solve quite complex tasks, e.g. [28], [30].

Aspects of TD learning in the context of spiking activity have been studied in [31]–[33]. However, the models developed in these studies do not perform the complete TD algorithm, which involves both prediction and control. Rao and Sejnowski demonstrate that in a two-neuron network, one neuron can learn to predict the firing times of the other [31], but the control aspect of TD learning is not addressed. The model presented by Farries and Fairhall includes an actor [32], but its decisions do not influence the state transitions. This is essentially a prediction task with a simplified TD error equal to the difference of the current reward and the average previous reward. The model proposed by Izhikevich uses a reward signal that is not equivalent to the TD error to solve a prediction task or to associate the presentation of a specific stimulus with one of two possible actions [33]. The fact that in each case the TD algorithm has been substantially simplified or reduced to just the prediction aspect is reflected in the simplicity of the tasks the models have been shown to solve. In these tasks either no reward is given at all [31] or a reward is given or withheld at the end of every episode [32], [33]. Such tasks are more akin to supervised learning paradigms, as the output of the network can be clearly identified as ‘right’ or ‘wrong’ for each decision.

Recently, we proposed the first spiking neuronal network model to implement a complete TD(0) implementation with both prediction and control, and demonstrated that it is able to solve a non-trivial task with sparse rewards [34]. However, in that model each synapse performs its own approximation of the TD error rather than receiving it in the form of a neuromodulatory signal as suggested by experimental evidence [2], [3]. We now present the first spiking neuronal model of an actor-critic TD learning agent that adapts its behavior on the basis of a dopaminergic signal dynamically generated by the network itself. We develop the model following a combination of top-down and bottom-up approaches. These terms can be interpreted in several different ways; see [35] for an analysis. Our interpretation is as follows: a top-down approach constructs a system to fulfill a desired function. In our case, we design synaptic plasticity rules that map to the update rules of temporal-difference learning whilst obeying reasonable biological constraints on the information available to the synapse. Conversely, a bottom-up approach to neuronal modeling integrates information from experimental analyses to generate a more complex system. Here, we integrate the known dynamical features of the dopaminergic activity with the sensitivity of cortico-striatal synapses to the presence of dopamine.

We show that dopamine-dependent plasticity relying on a dopaminergic signal with realistic firing rates can indeed realize TD learning. Our plasticity models depend on the global dopaminergic signal and the timing of pre- and post-synaptic spikes. Although the dynamics of the synaptic plasticity are constructed using a top-down approach to reproduce the key characteristics of the behavior-modifying updates of TD learning, we find a good agreement between the predictions of our plasticity models and experimental findings on cortico-striatal synapses. The discrepancies between the dopaminergic signal with realistic firing rates and the TD error result in a slightly modified TD learning algorithm with self-adapting learning parameters and an adapting offset. The parameters of our proposed synaptic plasticity models can be analytically mapped piecewise to the parameters of a classical discrete-time implementation of the TD algorithm for positive and small negative values of the TD error. We show that despite these modifications, the neuronal network is able to solve a non-trivial grid-world task with sparse positive rewards as quickly and as stably as the corresponding algorithmic implementation. The synaptic weights develop during the learning process to reflect the values of states with respect to their reward proximity as well as an optimal policy in order to maximize the reward. We demonstrate the consequences of the modifications to the learning algorithm on a cliff-walk task. The neuronal network cannot learn the task when the external rewards are purely negative. If the task includes both positive and negative rewards, the neuronal network can still learn it, but more slowly than the corresponding classical discrete-time algorithm and with a worse equilibrium performance. Our results support the hypothesis that negative rewards are mediated by different anatomical structures and neuromodulatory systems.

### Temporal-difference learning in the actor-critic architecture

In this article we focus on a specific variant of TD learning: the TD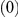 algorithm as implemented by the actor-critic architecture [36]. Here, we summarize the basic principles; a thorough introduction can be found in [1].

**Figure 1:**
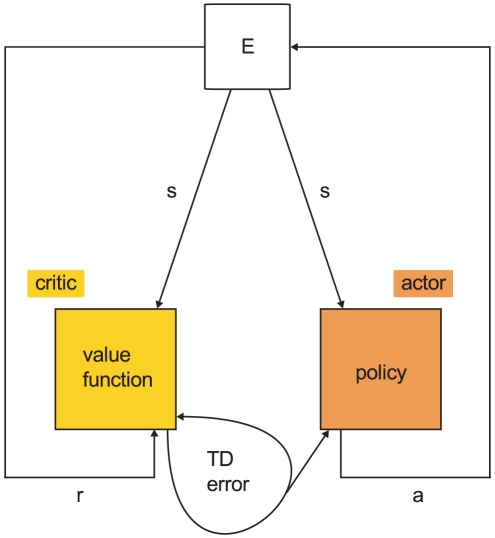
Actor-critic architecture. The environment (E) informs the critic and the actor about the current state (s). In addition, it transmits the current reward information (r) to the critic. The critic calculates based on the value function of the current and previous state and the reward information the TD error signal, which is used to update the policy and the value function of the previous state. The actor selects based on the policy of the current state an action (a), which is read out by the environment. (Figure adapted from [1]).

The goal of a TD learning agent, as for every reinforcement learning agent, is to maximize the accumulated reward it receives over time. The actor-critic architecture (see Fig. 1) achieves this goal by making use of two modules, the actor and the critic. The actor module learns a policy 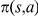, which gives the probability of selecting an action 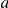 in a state 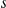. A common method of defining a policy is given by the Gibbs softmax distribution:
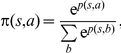
where 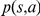 is known as the preference of action 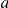 in state 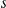 and the index 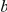 runs over all possible actions in state 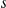.

The critic evaluates the consequences of the actor module's chosen actions with respect to a value function. Once learning has reached equilibrium, the value function 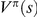 is the expected summed discounted future reward when starting from state 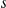 and following policy 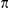. During the learning process only estimates 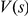 of the actual value function are available. The performance of the agent on a task is improved by making successive updates to the policy and the value function. These updates are usually formulated assuming a discretization of time and space: an error term 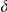 is calculated based on the difference in estimations of the value function when moving from one discrete state 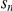 to the next, 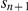:
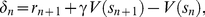
(1)where 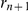 is the reward the agent receives when moving into state 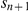 and 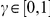 is a discount factor. This error signal 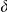, known as the TD error, is positive if the reward is greater than the expected discounted difference between 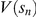 and 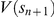, indicating that the estimate of 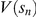 needs to be increased. Conversely, 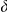 is negative if the reward is less than the expected discounted difference, indicating that the estimate of 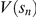 needs to be decreased. In the simplest version of TD learning, known as the TD(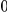) algorithm, the critic improves its estimate of 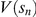 as follows:
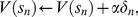
(2)where 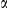 is a small positive step-size parameter. For a given policy and a sufficiently small 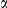, the TD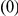 learning algorithm converges with probability 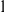 [37], [38]. Additionally, the preference of the chosen action 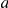 in state 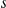 is adjusted to make the selection of this action correspondingly more or less likely the next time the agent visits that state. One possibility to update the preference in the actor-critic architecture is given by:
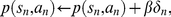
(3)where 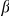 is another small step-size parameter. For the purposes of this manuscript, we shall refer to the calculation of the error signal and the update of value function and policy described above as the classical discrete-time TD(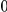) algorithm.

## Results

### Spiking actor-critic architecture

**Figure 2:**
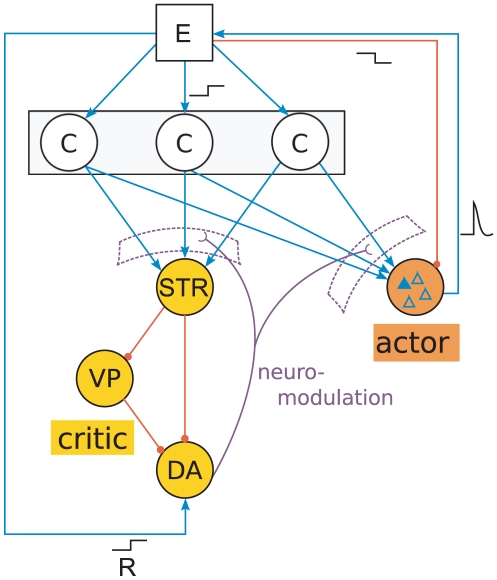
Neuronal actor-critic architecture generating and exploiting a dopaminergic TD error signal. The input layer of the neuronal network consists of pools of cortical neurons (C) representing state information. The critic module is composed of neurons in the striatum (STR), neurons in the ventral pallidum (VP) and dopaminergic neurons (DA). The direct pathway from the striatum to the dopamine neurons is delayed with respect to the indirect pathway via the neuron population in the ventral pallidum. The actor module consists of one neuron for each possible action. The neural network interacts with an environment (E). The environment stimulates the cortical neurons representing the current state with a DC input. Whichever action neuron fires first is interpreted by the environment as the chosen action for the current state. After an action has been chosen the environment inhibits the actor neurons for a short time period by a negative DC input. If the current state is associated with a reward, the environment delivers a reward signal (R) in form of a DC input to the dopaminergic neurons. The dopaminergic signal modulates as a global third factor the plasticity of cortico-striatal synapses and the synapses between cortex and actor neurons. Red lines; inhibitory connections, blue lines; excitatory connections, purple lines; dopaminergic signal. All neurons receive additional Poissonian background noise (not shown).

Fig. 2 illustrates the architecture of our actor-critic spiking network model implementing temporal-difference learning (see Introduction). All neurons in the network are represented by current-based integrate-and-fire neurons with alpha shaped post-synaptic currents. A tabular description of our model and its neuronal, synaptic and external stimulation parameters are given in Methods. The network interacts with an environment, which is implemented purely algorithmically for the purpose of this work. The input layer of the neural network represents the cortex; it encodes information about 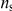 states, each represented by a population of 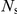 neurons. The environment stimulates the population associated with the current state of the agent with a constant DC input, causing the neurons to fire with a mean rate of 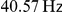; in the inactivated state the neurons fire on average with 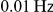. The low background rate in the inactivated state is chosen for the sake of simplicity in developing the synaptic plasticity dynamics, but is not a critical assumption of the model (see section “Synaptic-plasticity”). Each population in the cortex projects to the actor and critic modules.

As the focus of our study is the consequences of a realistic dopaminergic signal for temporal-difference learning rather than action selection, we keep the actor model as simple as possible. As in previous models [20], [34], [39], the actor module consists of 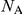 actor neurons, each corresponding to one action. The synaptic weights between the cortical and the actor neurons represent the policy in our model. Whichever action neuron fires first in response to the activation of the state neurons is interpreted by the environment as the chosen action (for a review of first-spike coding, see [40]). Immediately after an action has been chosen, i.e. after an actor neuron has spiked, the environment deactivates the previous state neurons and activates the neurons representing the new state resulting from the chosen action. At the same time the environment inhibits the actor neurons for a short time period 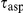, during which no further action can be chosen, allowing the cortical signal from a newly entered state to build up. For more sophisticated approaches to the action selection problem, see [41], [42].

**Figure 3:**
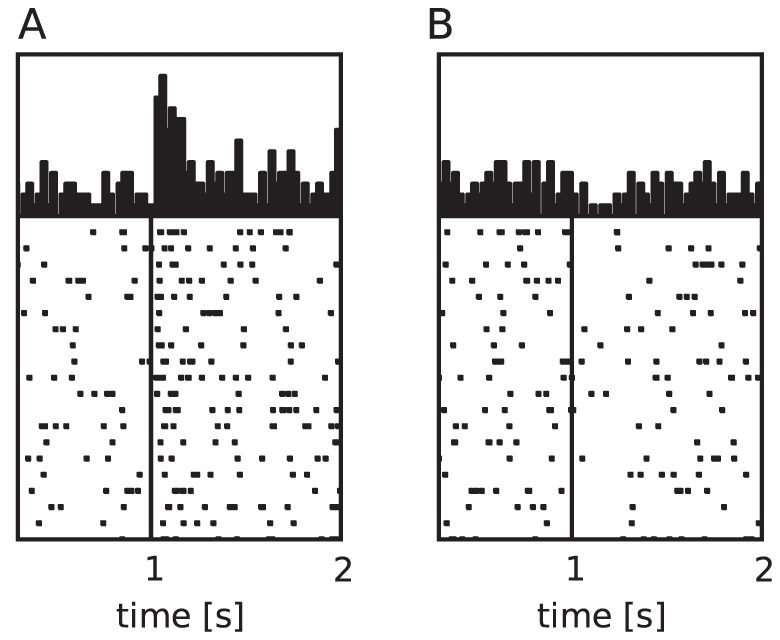
Spiking activity of one dopamine neuron in 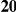 trials. (A) The agent moves from a state with cortico-striatal synaptic weights of 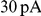 to a state with cortico-striatal synaptic weights of 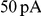 at 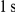, leading to a phasic increase in the dopaminergic activity. Each horizontal line in the lower panel shows the spike times of the dopamine neuron in one trial; the upper panel shows the histogram of the spiking activity over the 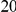 trials with a bin width of 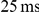. (B) As in (A), but here the agent moves from the higher valued state (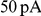) to the lower value state (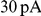) at 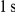 leading to a phasic decrease in the dopaminergic activity.

Two key experimentally observed features of the activity of the dopaminergic neurons are a constant low background rate with phasic activity with asymmetric amplitude depending on whether a reward is given or withheld [2]. As the basal ganglia dynamics generating this signal is unknown, we select the simplest possible network that generates these features; in general, multiple network configurations can produce the same dynamics [43]. We adapt the circuit model proposed in [18] to perform the role of the critic module, which is responsible for generating a temporal-difference error. The major model assumption here is that the weights of the synapses between the neurons representing a given state and the critic module encode the value of that state. The circuit connects a population of 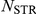 neurons representing the striatum, the input layer of the basal ganglia, to a population of 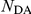 dopaminergic neurons directly and also indirectly via a population of 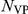 neurons representing the ventral pallidum. The direct and indirect pathways are both inhibitory. Consequently, the synaptic input from the striatum via the indirect pathway has a net excitatory effect, whereas the delayed striatal synaptic input via the direct pathway has an inhibitory effect on the dopamine neurons. This results in a phasic increase if the agent moves from a state with low cortico-striatal synaptic weights to a state with high weights (see Fig. 3) and a phasic decrease if the agent moves from a state with high cortico-striatal synaptic weights to a state with low weights. The length of the phasic activation is determined by the difference in the delays of the direct pathway 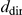 and the indirect one 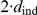. We have chosen 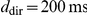 and 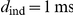 which results in a duration of the phasic activation similar to that observed experimentally (see Fig. 1 in [2]). If the agent enters a rewarded state, the dopamine neurons receive an additional DC stimulation from the environment starting 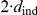 after the agent moves and lasting for the duration of the phasic activity, 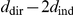. Assuming the cortico-striatal synaptic weights represent the value function, after each state transition the dopamine neurons integrate information about the current value function with a positive sign, information about the previous value function with a negative sign, and a reward signal. Thus all the information necessary to calculate a form of temporal-difference error is present (see Eq. (1)).

The 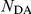 dopaminergic neurons project back and release dopamine into the extracellular space (Fig. 2 purple arrows) which modulates as a third factor the plasticity of the synapses between the cortex and the striatum and between the cortex and the actor neurons. Later in this section we develop synaptic plasticity models using a top-down approach to implement TD learning.

### Dopaminergic error signal

In this section we show that our network is able to generate dopaminergic activity with realistic firing rates and discuss its similarities to, and differences from, the classical discrete-time algorithmic definition of the TD error signal given in Eq. (1). It has been found that dopamine neurons fire with a low constant baseline activity (approx. 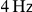 in rats [44], [45] and 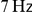 in monkeys [46]) as long as nothing unpredicted happens. This is known as the tonic activity of the dopaminergic neurons. For our model, this implies that the baseline firing rate should be independent of the strength of the cortical-striatal synapses associated with each state. This condition can be fulfilled in our architecture for an infinite number of configurations assuming linear relationships between the firing rates of the neurons in the striatum and the ventral pallidum; for a derivation of these relationships, see Supplementary Text S1. We select the simplest rate relationship with a linear coefficient of one. This relationship generates a constant baseline activity when 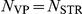 and the synaptic weights connecting the striatum to the dopamine neurons are equal in strength to the synaptic weights between the ventral pallidum and the dopamine neurons. For the parameters given in Methods the mean dopaminergic baseline firing rate in our network is approx. 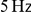, which is close to the experimentally observed stationary dopaminergic firing rate.

**Figure 4:**
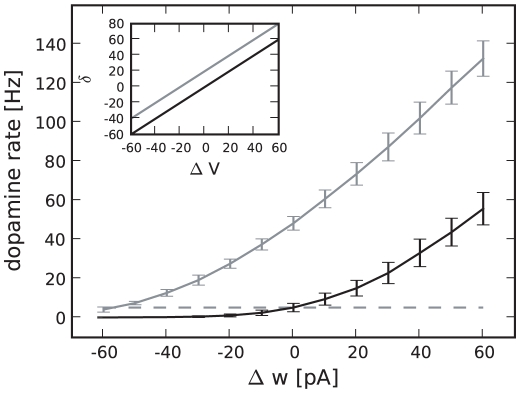
Amplitude of the phasic dopaminergic activity averaged over 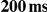 following a transition from state 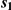 with cortico-striatal synaptic weights 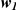 to state 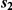 with cortico-striatal synaptic weights 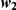 as a function of 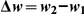. No external reward signal: black curve, external reward signal of 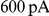: gray curve. The values of 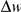 are chosen as 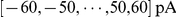; the data point for a specific weight difference is calculated as the amplitude of the dopaminergic rate excursion averaged over 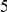 trials for each combination of 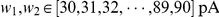 that results in that weight difference. Error bars indicate the standard deviation. The dashed black line indicates the dopaminergic base firing rate. Inset: discrete-time algorithmic TD error signal 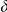 Eq. (1) as a function of 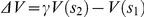 for 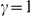. Reward 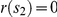: black curve, 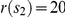: gray curve.

When the agent transits from one state to another, the dopamine neurons exhibit phasic activity lasting for around 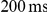 in accordance with durations found experimentally [47], [48], see Fig. 3. Fig. 4 shows the amplitude of phasic activity of the dopaminergic neurons after the agent transits from state 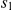 to state 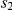 in dependence of the difference in the corresponding cortico-striatal synaptic weights 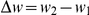. In accordance with experimental observation [46] the dopamine neurons show a continuum of firing rates lower than the baseline for outcomes that are worse than predicted (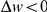) and higher than the baseline for outcomes better than expected (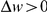). Likewise, entering a state with an unpredicted reward induces a phasic increase of activity. The amplitude of the phasic activity of the dopaminergic neurons therefore has similar properties to the algorithmic TD error signal given in Eq.(1). However, the properties of the dopaminergic signal deviate from the TD error 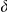 in the following points:

1. Due to the low baseline firing rate of the dopamine neurons, the dopaminergic signal does not have as large a dynamic range to represent negative errors as it has to represent positive errors
2. The phasic dopaminergic activity is a nonlinear function of the difference in cortico-striatal synaptic weights of successive states whereas the classical algorithmic TD error signal depends linearly on the difference in the value function for successive states
3. The slope of the phasic dopaminergic signal as a function of the difference in the cortico-striatal synaptic weights of successive states is greater when an additional reward signal is present
4. As the baseline firing rate is independent of the current striatal firing rate, i.e. the value of the current state, the amplitude of the phasic activity depends on the absolute difference between the value of two successive states 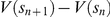 rather than the 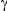-discounted difference 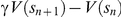

Point 2 arises due to the nonlinearities inherent in spiking neuronal networks, particularly at low rates (for a recent account see [49]). If a linear rate-based model was assumed, the amplitude of the phasic response would also vary linearly until an amplitude of 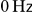 was reached for some negative value of 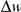. Similarly, the addition of the reward signal could only affect the offset of the curve in a linear rate-based model (point 3). A nonlinear rate-based model may well be able to capture these features, but in order to make the correct non-linear assumptions, the behavior of the system to be abstracted needs to be known first. A nonlinear dependence of the dopaminergic firing rate on the reward prediction error has recently also been observed experimentally [46]. As we show in the next subsection, point 4 can be compensated by introducing a discount factor in the synaptic plasticity dynamics. A 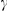-discounted difference can also be obtained if the dopaminergic rate is assumed to depend on the striatal firing rate. As this is not in accordance with experimental findings we do not make this assumption, however, a derivation of the relationship between the firing rates and 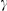 is derived in Supplementary Text S1.

### Synaptic plasticity

In order for the network model to realize TD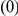 learning, the right synapses have to undergo the right changes in strength at the right time; this is also known as the credit assignment problem [1]. Here, we derive synaptic plasticity dynamics in a top-down fashion for the cortico-striatal synapses and the synapses between the cortical populations and the actor module representing the value function and the policy respectively. In the classical TD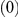 algorithm, when the agent transits from state 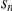 into state 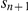, only the value 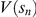 and preference 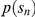 of the most recently exited state 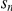 are updated (see Eq. (2) and Eq. (3)).

**Figure 5:**
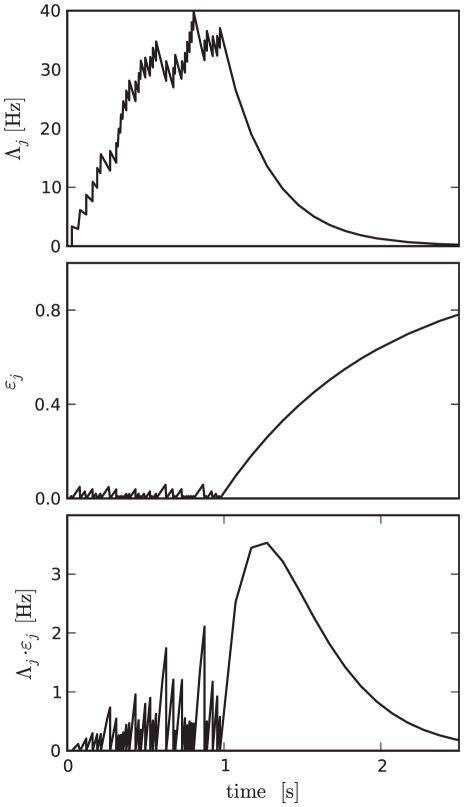
Pre-synaptic activity trace 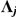 (top), pre-synaptic efficacy trace 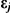 (middle) and their product 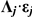 (bottom) as functions of time. The agent enters the state represented by the pre-synaptic neuron 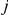 at time 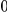 and leaves the state at 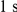.

For a synapse to implement this feature it requires a mechanism that enables plasticity for a short time period after the agent has left the state associated with the pre-synaptic neuron. This situation is characterized by the pre-synaptic rate being initially high and then dropping, as the population of cortical neurons associated with a state is strongly stimulated when the agent is in that state and weakly stimulated otherwise. An appropriate dynamics can be constructed if the synapse maintains two dynamic variables driven by the spikes of the pre-synaptic neuron 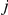 as originally proposed in [34]: a pre-synaptic activity trace 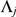 and a pre-synaptic efficacy trace 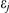:
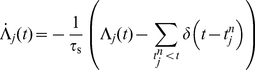
(4)

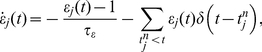
(5)where 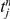 denotes the 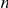
th spike of the pre-synaptic neuron 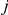. The pre-synaptic activity trace is an approximation of the pre-synaptic firing rate; it is incremented at every pre-synaptic spike and decays to 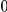 with a time constant 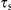 (see top panel of Fig. 5). To restrict the plasticity to the period immediately following a state transition, we assume a value of 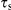 such that the activity trace decays to zero before the agent performs a further state transition. Efficacy traces as defined in Eq.(5) have previously been postulated as part of a spike-timing dependent plasticity model that accounts for data obtained from triplet and quadruplet spike protocols [50]. The efficacy trace is set to 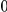 at every pre-synaptic spike and relaxes exponentially to 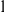 with a time constant 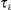 (Fig. 5, middle panel). This time constant is assumed to be large such that 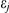 is small in the presence of pre-synaptic activity. When the agent is in the state associated with neuron 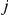, 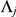 is high and 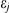 is close to zero. When the agent leaves the state, 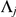 relaxes to 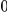 and 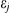 relaxes to 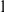. A product of the two traces is therefore close to 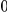 at all times except for the period shortly after the agent leaves the state associated with neuron 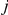 (Fig. 5, bottom panel). Therefore, a synaptic plasticity dynamics proportional to 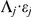 ensures that the right synapses are sensitive to modifications at the right time to implement TD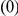 learning.

This simple relationship only holds for a very low rate in the inactive state. If the firing rate of cortical neurons in the inactive state were higher, then the product 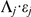 would be non-negligible at all times, resulting in permanent sensitivity of the synapse to irrelevant fluctations in the dopamine signal. Of course, this could be compensated for without altering the functionality by requiring 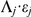 to exceed a threshold, or by adopting a triphasic approach based on successive pre-synaptic activity thresholds as in our earlier work [34]. The low rate therefore does not constitute a requirement for our model. However, to avoid additional factors in the plasticity dynamics, we prefer to keep the rate relationships as simple as possible.

In TD learning the value function and the policy are both updated proportionally to the TD error (see Eq. (2) and Eq. (3)) which in our network model is signalled by the deviation of the dopaminergic firing rate from its baseline. For the sake of simplicity we model the dopamine concentration 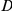 as the superposition of the activity traces of all dopaminergic neurons:
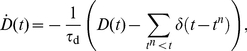
(6)where 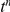 is the 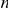
th dopamine spike and 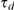 is a time constant. This simplified model captures the experimentally observed feature that the concentration of dopamine is dependent on the firing times of the dopaminergic neurons [51], [52]. Moreover, we set 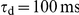 in agreement with experimental findings on the dopamine uptake time in the striatum [51]. A more sophisticated approach to modelling the extracellular dopamine concentration can be found in [52]. A suitable synaptic plasticity dynamics between a cortical neuron 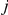 and a striatal neuron 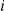 to implement value function updates is therefore given by:
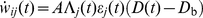
(7)where 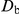 is the baseline concentration of dopamine and 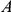 is a learning rate parameter.

As discussed in the previous section, one difference between the dopaminergic signal as generated by our network model and the TD error is that the dopaminergic firing rate depends on the total value of the current state, rather than the 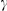-discounted value (compare Eq.(2)). However, it is possible to compensate for this discrepancy in the following way. The firing rate of the striatum population expresses the value of the current state, as the value function is encoded by the cortico-striatal synaptic weights. For a given cortico-striatal synapse, the current state value can therefore be approximated by a post-synaptic activity trace as defined in Eq. (4) with a time constant 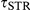, which can be chosen quite arbitrarily. We therefore include a term in Eq. (7) proportional to the post-synaptic activity trace 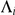:
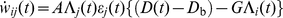
(8)where 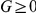. In our numerical simulations we assume a plasticity dynamics at the cortico-striatal synapses as given by Eq. (8).

During the short period after a transition from 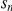 to 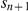, the cortico-striatal synapses associated with state 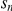 are sensitive to modification. As discussed in the previous section, the dopaminergic signal depends nonlinearly on successive reward predictions encoded in the cortico-striatal synaptic weights, whereas the TD error is a linear function on the value function of successive states. Furthermore the slope of the non-linear function depends on the magnitude of any external reward. This means that it is not possible to define a single mapping from the units of synaptic weights to the units of the value function that holds for all values of 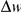 and all rewards, as in our previous study [34]. However, it is possible to generate a piecewise mapping by approximating the nonlinear function for a given reward signal in a given range of 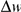 by a linear function.

The mapping (Eq. (11)) is derived in detail in the Supplementary Text S2 and consists of two steps. First, the synaptic plasticity dynamics is integrated to calculate the net change in the mean outgoing synaptic weight of the neurons associated with a state 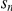 when the agent moves from 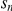 to 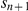. Second, the net weight change is converted from units of synaptic weight to units of the value function according to the linear relationships:
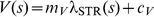
(9)

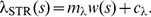
(10)where 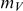 is a proportionality parameter mapping the mean striatal firing rate 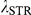 to the units of the value function 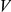 and 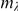 is a proportionality factor mapping the mean cortico-striatal weights of a state 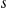 to the mean striatal firing rate. For our choice of parameters (see Methods) Eq. (10) is fulfilled in the allowed range for the cortico-striatal weights with 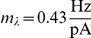 and 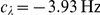.

Within a given range of 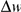, the mean net weight change of the synapses immediately after transition out of 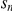 corresponds to a slightly modified version of the classical discrete-time value function update with an additional offset 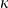:
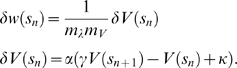
(11)


The learning parameters 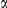 and 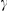 of the equivalent TD(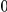) algorithm and the offset 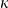 depend on the synaptic parameters 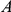 and 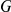 as defined above. They additionally depend on the slope 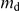 and intercept 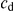 of the linear approximation of the dopaminergic signal:
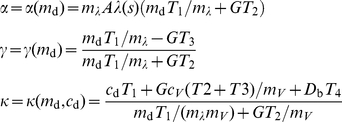
(12)The constants 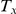 depend on the synaptic time constants; see Supplementary Text S2 for the definitions.

Because 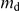 and 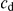 are dependent on the range of 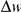 and the direct current applied to the dopamine neurons, the weight update 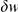 can be interpreted as a TD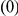 learning value function update with self-adapting learning parameters and a self-adapting offset that depend on the current weight change and reward. The greater the difference between the mean synaptic weights of successive states 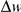, the higher the learning rate 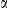 and discount factor 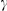. For the parameters used in our simulations, a range of 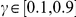 can be realized by a range of 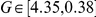. A choice of 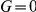 results in a discount factor 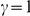. For a specific choice of 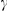, the learning rate 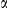 can be determined by the synaptic parameter 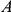. For 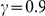, the range 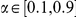 can be realized by the range 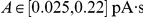. As 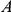 and 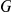 can be chosen independently, all possible combinations of 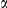 and 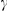 can be realized.

If the current state is rewarded, the offset 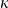 is a 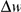-dependent analog to the reward in the TD error Eq. (1). Otherwise, for an appropriate choice of parameters (see Methods) 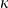 is always smaller than 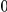 and has no analog in classical TD learning.

Self-adjusting parameters have also been implemented in other three-factor learning rules such as the one in [53] based on the meta-learning algorithm proposed in [54]. In contrast to meta-learning, in our model the values of the parameters do not adjust themselves to optimal parameters for a given task but vary according to the difference between the estimated values of successive states, 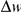, and the current reward value. The range of possible learning parameters for a given 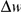 and reward value depends on the current choice of synaptic parameters 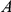 and 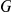, which can be set arbitrarily. However, meta-learning could be an additional mechanism that adjusts the parameters 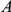 and 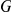 to optimal values for a given task.

The variable parameters suggest a similarity with value learning, another learning algorithm similar to TD but with a variable discount rate [55]. However, in value learning the discount rate changes over time: it is lowest immediately after an unconditioned stimulus and increases in between them, making the algorithm more sensitive to long term rewards. In our model the learning parameters do not depend on time but on the current reward and the difference in successive reward predictions encoded by 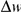.

Similarly to the update of the value function, in the classical discrete-time TD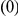 algorithm only the policy for the recently vacated state is updated. As described earlier in this section, in the neuronal architecture an action is chosen by the first spike of an action neuron. Therefore an appropriate plasticity dynamics for the synapse between a cortex neuron 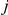 and an actor neuron 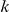 is given by
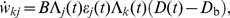
(13)where 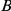 determines the learning speed, and 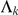 is a post-synaptic activity trace as defined in Eq. (4) with time constant 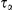. The choice of post-synaptic time constant is not critical, but the activity trace should decay within the typical time an agent spends in a state in order to be selective for the most recently chosen action. Unlike the cortico-striatal synapses described above, the lack of 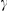-discounting in the dopamine signal cannot be compensated for by the addition of an additional local term in the synaptic plasticity dynamics. This is due to the fact that the post-synaptic activity here represents whether the encoded action was selected rather than the value function of the next state as in the previous case. Information about the value of the new state could only arrive at the synapse through an additional non-local mechanism.

In order to ensure the agent continues to occasionally explore alternative directions to its preferred direction in any given state, we restrict the synaptic weights of the synapses between the cortex and the actor neurons to the range 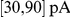. This results in a maximal probability of 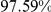 and a minimal probability of 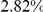 for any movement direction in any state (see Supplementary Text S2 for a mapping of synaptic weights to probabilities).

The parameters for synaptic plasticity models used in our study are summarized in Methods.

### Comparison of predictions of the synaptic plasticity models with experimental results

The proposed cortico-striatal synaptic plasticity dynamics Eq. (8) depends on three factors: the pre-synaptic firing rate, the post-synaptic firing rate and the dopamine concentration. For cortico-striatal synapses the effect on the plasticity of each of these factors has experimentally been studied in vivo and in vitro (see [9] for a review). The long-term effects found on average across studies are summarized in column six of Table 1. These results show that in order to induce any long lasting changes in synaptic plasticity, a conjunction of pre- and post-synaptic activity is required. Early studies on the effect of conjoined pre-synaptic and post-synaptic activity on the cortico-striatal plasticity reported exclusively long term depression (LTD). More recent studies have shown that long term potentiation (LTP) can also be obtained under some circumstances. The expression of LTP or LTD seems to depend on methodological factors such as the age of the animal, the location of the neuron and the stimulating electrode and the stimulus parameters [9]. Although in these studies it is assumed that dopamine is not involved, it cannot be ruled out as cortico-striatal high frequency stimulation causes dopamine release [56]. The main findings resulting from studies involving all three factors can be summarized in the following three-factor rule [57]: under normal and low dopamine concentrations, the conjunction of pre- and post-synaptic activity leads to LTD, whereas a large phasic increase in dopamine concentration during pre- and post-synaptic activity results in LTP.

**Table 1:** Theoretical predictions of cortico-striatal synaptic plasticity dynamics as functions of pre-synaptic activity, post-synaptic activity, and dopamine concentration in comparison with the average experimental findings across studies on long-term effects in synaptic plasticity.

pre	post	dopa	theoretical predictions 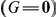	theoretical prediction 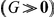	experimental results
0	0	0	-	-	-
1	0	0	-	-	-
0	1	0	-	-	-
0	0	1	-	-	-
1	1	0	-	LTD	LTD (LTP)
1	0	1	LTD 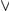 LTP	LTD 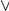 LTP	-
0	1	1	-	-	-
1	1	1	LTD 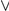 LTP	LTD 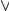 LTP	LTD 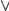 LTP

The predictions are based on eq:value function weight update for 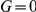 and 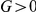, corresponding to discount factors 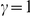 and 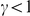, respectively; the experimental findings on [9]. A 1 in the first three columns denotes an active influence, whereas a 0 indicates that the corresponding activity is not involved in the synaptic changes. The symbol 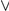 indicates that either LTD or LTP occurs depending on the concentration of dopamine; the symbol - denotes an absence of long-term changes in the synaptic weights.

The predictions of the cortico-striatal synaptic dynamics given by Eq. (8) for the various permutations of pre- and post-synaptic activity and dopamine concentration are summarized in column 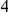 (for 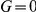, corresponding to 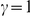) and column 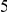 (for 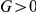, corresponding to 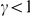) of Table 1. We assume that a value of 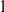 in the first three columns denotes recent activity; due to the time constants of the activity traces this activation is still perceptible from the point of view of the synapse and can thus be assumed to have an active influence on plasticity. Assuming the baseline dopamine concentration 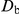 only changes on a long time scale, experiments involving no particular manipulations of the dopamine concentration (denoted by 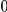 in Table 1) will be characterized by 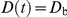. The plasticity dynamics Eq. (8) predicts LTD for an active influence of pre- and post-synaptic activity, 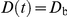 and 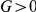 in accordance with the majority of the experimental findings; for 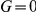 no change in synaptic strength is predicted.

**Figure 6:**
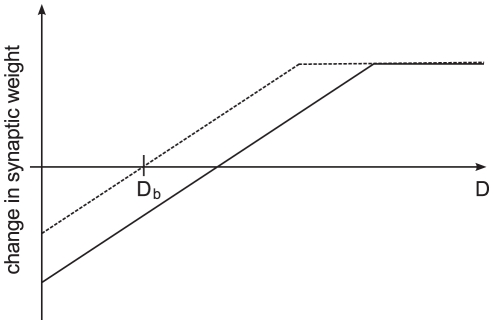
Change in strength of cortico-striatal synapses predicted by Eq. (8) as a function of the dopaminergic concentration 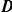 assuming a conjunction of pre- and post-synaptic activity for 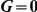 (dashed line) and 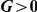 (solid line). For 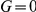, the change from LTD to LTP occurs at 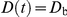, whereas for 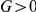 the switch occurs at a higher concentration of dopamine.

Furthermore, Eq. (8) predicts that for simultaneous influence of pre- and post-synaptic activity, the direction of the synaptic change depends on the concentration of dopamine. For 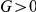 normal (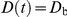) as well as low dopamine concentration 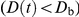 results in LTD (see Fig. 6), while a large phasic increase in the dopamine concentration 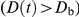 results in LTP. For 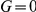 the change from LTD to LTP occurs at 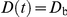, resulting in no change in synaptic strength under normal dopamine concentration in contrast to the experimental findings. The theoretical model makes additional predictions in this case that go beyond the presence or absence of activity and the direction of change. Due to the timing sensitivity of the plasticity dynamics given in Eq. (8), a weak synaptic weight change is predicted if the activity of the pre-synaptic neuron overlaps with the activity of the post-synaptic neuron in the presence of dopamine and a strong change if the pre-synaptic activity precedes the post-synaptic activity. Such a dependency on timing involving extended periods of activation have so far not been tested experimentally. However, protocols involving individual spike pairs have revealed comparable effects; for a review, see [58].

The greatest difference between our predictions and the experimental findings is that a simultaneously active influence of pre-synaptic activity and dopamine is sufficient to induce LTD or LTP in the absence of post-synaptic activity. However, this is quite an artificial case as pre-synaptic activity always generates post-synaptic activity in our network model dynamics. The behavior of the model could be brought into better alignment with the experimental data by adding additional complexity. For example, a multiplicative Heaviside function that evaluates to one when the post-synaptic activity exceeds a certain threshold would eliminate the generation of LTP/LTD in the absence of post-synaptic activity without altering the functionality of our model. As the plasticity dynamics was derived to fulfil a particular computational function rather than to provide a phenomenological fit to the experimental data, we prefer to avoid this additional complexity. Apart from this case, our predictions on the direction of cortico-striatal plasticity under the active conjunction of pre- and post-synaptic activity for 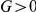 are in good agreement with experimental findings.

### Grid-world task

**Figure 7:**
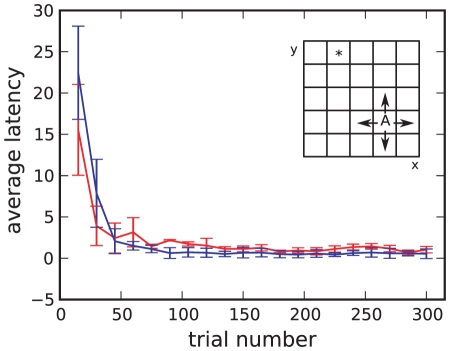
The grid-world task. Average latency in reaching the reward state and standard deviations over 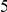 runs for the neuronal network model with optimized parameters 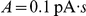, 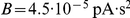, 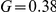 and reward 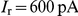 (red curve) and the corresponding classical discrete-time algorithmic TD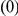 implementation with 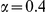, 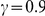, 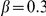 and reward 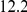 (blue curve). Each data point shows the average latency over 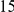 successive trials. Inset: grid-world environment consisting of 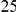 states. Only the state marked with an asterisk is rewarded. In each state the agent (A) can choose between 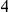 directions (indicated by the arrows). Once the rewarded state has been found, the agent is moved randomly to a new starting position.

As in our previous study [34], we tested the learning capability of our neuronal network model on a grid-world task, a standard task for TD learning algorithms. In our variant of this task, the grid consists of 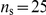 states arranged in a five by five grid (see inset of Fig. 7). The agent can choose between four different actions (south, north, east, west) represented by 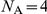 actor neurons. If the agent chooses an action which would lead outside the grid world, the action does not lead to a change in its position. Only a single state is rewarded; when the agent enters it a direct current with amplitude 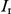 is applied to the dopaminergic neurons corresponding to the real-valued reward sent to the critic module in a classical discrete-time TD algorithm (see Introduction). After the agent has found the reward and selected a new action, it is moved to a new starting position that is chosen randomly and independently of the selected action. This is therefore a continuing task rather than an episodic task, as there are no terminal states. To maximize its reward, the agent must find the reward from random starting positions in as few steps as possible. The difficulty of the task is that the agent has to learn a series of several actions starting from each state in which only the last one results in a reward. The grid world task is useful to visualize the behavior of a learning algorithm but is not intended to represent physical navigation task, as spatial information is not taken into consideration (e.g. exploiting the knowledge of which states are neighbors).

To evaluate the performance of our model on the grid-world task, we separate the ongoing sequence of states and actions into trials, where a trial is defined as the period between the agent being placed in a starting position and the agent reaching the reward state. We measure the latency for each trial, i.e. the difference between the number of steps the agent takes to reach the reward state and the minimum number of steps required to reach the reward state for the given starting position. To provide a comparison, we also measure the performance of a classical discrete-time TD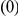 learning algorithmic implementation with corresponding parameters. The specification of the discrete-time implementation is obtained by mapping the synaptic parameters to the discrete-time parameters for 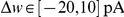 and determining the corresponding reward via a search algorithm (see Supplementary Text S2).

Fig. 7 shows the evolution of latency on the grid-world task for the neuronal network model with optimized parameters and the discrete-time algorithmic implementation with corresponding parameters. Within the first 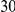 trials the latency of the neuronal network model drops from around 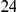 steps to 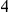 steps. After 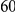 trials the agent has learnt the task; the latency is always below 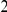 steps. The learning speed and the equilibrium performance of the neuronal network model are as good as those of the corresponding discrete-time algorithmic implementation. The performance of the discrete-time algorithmic implementation does not deteriorate if a discount factor 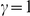 is assumed for the updates to the policy in correspondence with the synaptic plasticity dynamics given by Eq. (13) (data not shown).

As discussed in section “Synaptic-plasticity”, we impose hard bounds on the weights of the synapses between the cortex and the actor to ensure that for a given state, no action becomes either impossible or certain. For this task, it turns out that the lower bound is not necessary; restricting the weights to the range 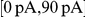 results in a similar learning performance (data not shown). However, the upper bound is necessary for the stability of the system. In the absence of an upper bound, synaptic weights between the cortex and all action neurons other than south increase to unbiological levels. This runaway behavior is detrimental to the learning process; in 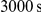 the agent only locates the rewarded state 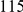 times, a factor of 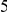 fewer than for the bounded learning agent.

**Figure 8:**
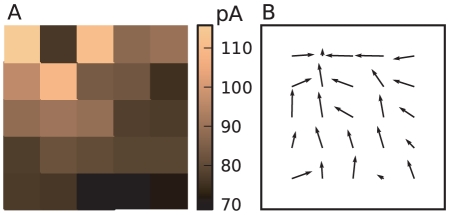
Average value function and policy over 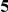 runs for the neuronal network model after 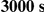 simulation of biological time corresponding to around 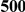 trials. (A) Value function. Each square represents the mean synaptic weight between the cortical neurons representing the associated state and the striatal neurons of the critic module (see Fig. 2). (B) Policy. The arrows indicate the preferred direction for each state given by the mean synaptic weights between the cortical neurons representing the associated state and the actor neurons.

In our model, all cortico-striatal synaptic weights as well as all synaptic weights between the cortex and the actor neurons are initialized with the same value. This corresponds to all states being estimated at the same value and all possible directions of movement from each state being equally preferred. Fig. 8A shows the value function encoded in the mean synaptic cortico-striatal weights associated with each state after the task has been learnt. A gradient towards the rewarded state can be seen, showing that the agent has learnt to correctly evaluate the states with respect to their reward proximity. In order to represent the policy, we mapped the synaptic weights between cortex and actor neurons to the probabilities of choosing each action (see Supplementary Text S2). Fig. 8B shows the preferred direction in a given state after the task has been learnt indicated by the arrows. The x-component of an arrow 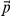 in a state 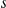 gives the difference between the probabilities 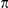 of choosing east and west, the y-component the difference between the probabilities of choosing north and south:
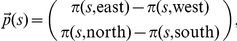
After the task has been learnt the agent tends to choose actions that move it closer to the rewarded state. These results show that not only can our model perform the TD(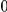) algorithm, but that its parameters can be successfully mapped to an equivalent classical discrete-time implementation. Despite the inherent noisiness of the neuronal network implementation, it learns as quickly and as well as a traditional algorithmic implementation.

### Cliff-walk task

In the previous section we demonstrated the ability of the spiking neuronal network model to solve a reinforcement learning problem with sparse positive reward. However, due to the asymmetry of the dopaminergic signal, it is to be expected that differences between the neuronal network model and a standard TD learning algorithm become more apparent in tasks where learning is driven by negative rewards. In this section we study the learning performance of the spiking neuronal network model in tasks with negative rewards and investigate the consequences of the modified TD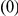 learning algorithm implemented by the neuronal network.

**Figure 9:**
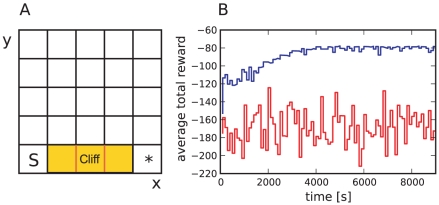
The cliff-walk task. (A) The environment consists of 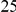 states. The agent starts each trial in the start state, marked with S and ends at the goal state, marked with an asterisk. The three states between the start state and the goal state represent the cliff. When the agent either moves into the cliff state or the goal state it is sent back to the start state. In a first variant of this task the agent never receives positive rewards. It receives a large negative reward for moving into the cliff and a smaller negative reward in all other states except the start and goal states, which have a reward of zero. In a second variant of this task the agent receives a positive reward for moving into the goal state and a negative reward when for moving into the cliff; in all other states the reward is zero. (B) Performance on the first variant of the cliff-walk task. Total reward in 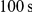 bins averaged over 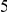 runs for the neuronal network model (red curve) and the discrete-time algorithmic TD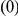 learning implementation (blue curve).

An appropriate task to discriminate between the standard and the modified TD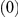 algorithms is the cliff-walk task [1]. In our version of this task, the cliff-walk environment consists of 25 states with five special states: a start state in the lower left, a goal state in the lower right and three cliff states in between the start and the goal state (see Fig. 9A). When the agent moves into a cliff state (i.e. falls off the cliff) a negative direct current with amplitude 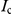 is applied to the dopaminergic neurons, corresponding to a negative reward value in a traditional TD learning algorithm. In the cliff states and the goal state, the agent is sent back to the start state regardless of the next action selected. As before, we treat the task as a continuous one, i.e. the synaptic weights representing the value function and the policy are continuously updated, even when the agent is sent back to the start state.

In a first variant of this task, a smaller negative direct current 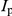 is applied to the dopamine neurons in all non-cliff states except the start and goal states, where the reward is zero. Thus, the agent only receives negative rewards from the environment. Setting 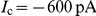 and 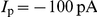 corresponds to setting a negative reward of 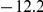 in the cliff states and 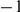 in all other states except the start and goal states for the discrete-time algorithmic TD(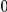) agent.

Fig. 9B shows the total reward received by the neuronal agent and the traditional algorithmic agent, summed in bins of 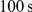 and averaged over 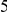 runs. All parameters are set as for the grid-world task. The traditional TD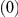 learning agent improves its performance rapidly. After approx. 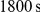 the average reward over 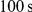 is always above 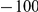. The performance continues to improve up to 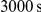, after which the average reward saturates at around 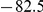. Unlike the grid-world task, the neuronal agent does not improve its performance even after 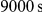. During this time the neuronal agent reaches the goal state on average only 30 times. In the same period the traditional agent reaches the goal state on average more than 700 times. Similarly, the average number of times the neuronal agent falls off the cliff is around 660, whereas the traditional agent makes this mistake on average less than 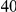 times. These results demonstrate that although the neuronal agent performs as well as the traditional discrete-time agent on the grid-world task, the traditional agent can learn the cliff-walk task with purely negative rewards and the neuronal agent cannot. This is due to the fact that the true underlying optimal value function is negative for this variant of the task, as the expected future rewards are negative. Thus, the synaptic weights representing the value function all reach their minimal allowed values and do not allow the agent to distinguish between states with respect to their reward proximity.

In a second variant of this task the agent receives a positive reward in the form of a direct current with amplitude 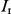 applied to the dopaminergic neurons when it reaches the goal state. The reward in all other states except the cliff and goal states is zero. For the purposes of analysis, the end of a trial is defined by the agent reaching the goal state, regardless of the number of times it falls off the cliff and is sent back to the start state.

**Figure 10:**
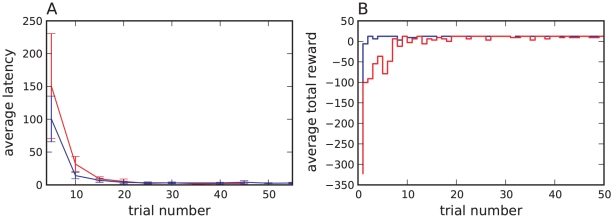
Performance on the second variant of the cliff-walk task. (A) Average latency in reaching the goal state and standard deviations over 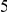 runs for the neuronal network model with 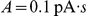, 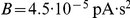, 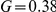, positive reward 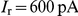 and negative reward 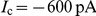 (red curve) and the corresponding classical discrete-time algorithmic TD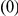 implementation with 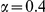, 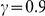, 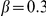, positive reward 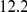 and negative reward 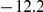 (blue curve). Each data point shows the average latency over 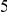 successive trials. (B) Total reward in each trial averaged over 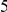 runs for the neuronal network model (red curve) and the discrete-time algorithmic TD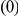 learning implementation (blue curve).

Fig. 10A shows the development of the latency on the cliff-walk task for the neuronal network model and the discrete-time algorithmic implementation, both with the same parameters used in the grid-world task. The cliff-walk task can be learnt much faster than the grid-world task, as the start state is not randomized, so the agent only needs to learn a good policy for the states around the cliff and the goal. The neuronal network model learns the task more slowly than the discrete-time algorithmic implementation, requiring around 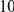 trials and 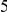 trials, respectively. The average latency after learning is slightly higher for the traditional agent (approx. 3) than for the neuronal agent (approx. 2.3). However, this does not mean that the neuronal agent has learned a better strategy for the task, as can be seen in the average total reward per trial shown in Fig. 10B. For the traditional algorithm, the summed reward after learning is equal to the reward of the goal state in almost every trial, demonstrating that the agent has learnt to completely avoid the cliff. The average reward received by the neuronal agent deviates much more frequently from the maximum, which shows that the neuronal agent still selects actions that cause it to fall off the cliff.

As for the grid-world task, it turns out that the upper bound on the weights of the synapses between the cortex and the actor neurons is necessary for the stability of the system but the lower bound is not. In the absence of an upper bound, the agent still initially learns the task within about 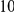 trials. However, the synaptic weights increase to unbiologically high values after approximately 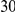 trials, which causes the task to be unlearned again. In contrast, the absence of a lower bound on the synaptic weights does not affect the learning performance (data not shown).

**Figure 11:**
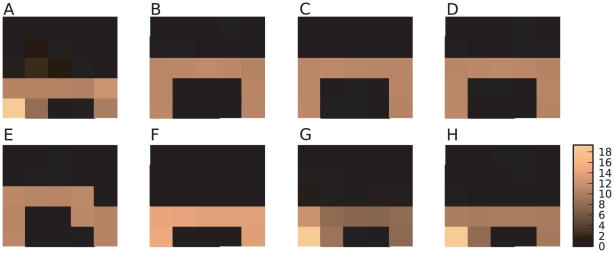
Learned strategies in the first variant of the cliff-walk task. Color indicates the number of visits the agent makes to that state as a percentage of 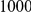 visited states in one run after learning is complete. (A) Neuronal agent. (B) Traditional TD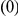 learning agent. (C) Modified discrete-time TD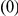 learning agent with a minimal TD error 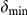. (D) Modified TD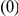 learning agent with a lower and an upper bound in the value function. (E) Modified TD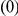 learning agent with a discount factor present only in the value function. (F) Modified TD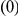 learning agent with self-adapting parameters and an additional offset. (G) Modified TD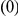 learning agent with adapting parameters and offset in addition to a bounded value function. (H) Modified TD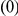 learning agent implementing all limitations studied individually in (C–F).

The differences in the behavior learned by the traditional and neuronal agents are also evident in Fig. 11, which shows for one run the relative frequencies with which each state is visited after the performance has reached equilibrium. For this purpose, we assume an agent to have reached equilibrium performance once it has visited 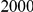 states. While the traditional agent (Fig. 11B) has learnt to avoid the cliff altogether and chooses a safe path one row away from the cliff, the neuronal agent (Fig. 11A) typically moves directly along the edge of the cliff and in some trials falls off it. The differences in the strategies learned by the traditional and the neuronal agents account for the finding that the neuronal agent exhibits a shorter average latency but a lower average reward per trial than the traditional discrete-time TD(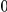) agent.

As discussed in section “Synaptic-plasticity” and derived in detail in the Supplementary Text S2, the neuronal network implements a modified TD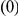 learning algorithm with self-adapting learning parameters 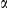 and 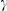, and a self-adapting additional offset (see Eq. (11) and Eq. (12)). Furthermore, a discount factor 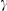 is only present in the value function update and not in the policy update. Another constraint of the neuronal network is that there is a natural lower bound in the dopaminergic firing rate, so there is a limited representation of negative temporal-difference errors. Similarly, the synaptic weights encoding the value function and the policy have lower bounds and are thus limited in their ability to encode negative values for states.

To analyze the consequences of these modifications from the traditional learning method, we implement modified versions of the traditional discrete-time TD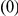 learning algorithm incorporating the various modifications present in the neuronal network model. The learned strategies are visualized in Fig. 11C–H. In all variants as well as in the original discrete-time TD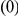 learning algorithm, we restrict the maximal and the minimal values for the action preferences 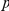 to the range 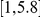. This results in the same maximum probability of choosing an action as given in the neuronal network by the bounds on the synaptic weights representing the policy. In all versions the parameters are set according to our derived mapping; the units of the synaptic weights are mapped into the units of the value function according to Eq. (9) for 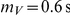 and 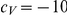.

In the first version, a lower bound 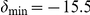 is applied to the TD error, thus limiting the system's ability to express that an action led to a much worse state than expected (Fig. 11C). In the second version the values of the value function are bounded to a minimal value function of 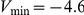 and a maximal value function of 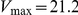 (Fig. 11D). Neither version results in a different strategy on the cliff-walk task from that learned by the traditional algorithm without modifications (Fig. 11B). A minor difference can be seen for the third version (Fig. 11E), which applies a discount factor 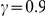 to the updates of the value function but not to those of the policy. We can therefore conclude that none of these modifications in isolation substantially alters the strategy learned for the cliff-walk task by the traditional TD(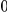) algorithm. The fourth version incorporates self-adapting learning parameters and an additional self-adapting offset in the TD error as given by Eq. (11) and Eq. (12). The mapping results in the following parameter sets for different external reward values: 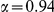, 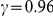 and 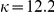 for the goal state, 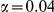, 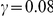 and 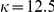 for the cliff states and 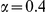, 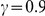 and 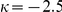 for all other states. This modification results in a strategy that is much more similar to that developed by the neuronal system, in that the agent typically walks directly along the edge of the cliff (Fig. 11F). Unlike the neuronal system, the modified TD(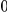) algorithm does not select actions that cause it to fall off the cliff. This can be clearly seen as the cliff states are not visited at all and all the states on the path are equally bright, indicating that the agent is only returned to the start state at the successful end of a trial. The key component of the modification is likely to be the additional offset: a similar strategy is learned by the traditional TD learning agent in an altered version of the cliff-walk task, in which each state other than the goal and the cliff states is associated with a negative reward equivalent to the offset (data not shown).

By combining the modifications, the strategy of the neuronal agent is recovered. Fig. 11G shows the strategy learned by a TD learning algorithm with self-adapting learning parameters and offset and with the value function restricted to the range 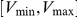. In this case, the agent mostly chooses the path closest to the edge of the cliff, but occasionally selects actions that cause it to fall off. Additionally enforcing a lower bound on the TD error and applying the 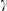-discount to the value function updates only do not cause any further alterations to the learned strategy (Fig. 11H).

These results show that whereas the neuronal agent cannot learn a task with purely negative rewards, it can learn a task where external negative rewards are applied when the underlying optimal value function is positive. However, even in this case the neuronal agent learns more slowly than a traditional agent and attains an inferior equilibrium performance. For the cliff-walk task, it is the self-adapting parameters and additional offset which contribute the most to the difference in the strategies learned by the neuronal and traditional agents. The bounds imposed on the value function in the modified TD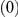 algorithm contribute second most, whereas the lower bound on the TD error and the absence of 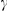-discounting on the policy updates do not play major roles.

## Discussion

We have presented the first spiking neuronal network model of an actor-critic temporal-difference learning agent that simultaneously accounts for multiple experimental results: the generation of a dopaminergic TD error signal with realistic firing rates, and plasticity dynamics in accordance with experimental findings with respect to pre-synaptic activity, post-synaptic activity and dopamine. The predictions of our plasticity dynamics are furthermore compatible with those of a recently proposed kinetic model of cortico-striatal synaptic plasticity [59]. The good agreement of the predictions of the proposed plasticity dynamics with experimental findings is particularly surprising, as we constructed the dynamics of the synaptic plasticity to result in TD learning using a top-down approach. The agreement between the synaptic dynamics derived from computational principles and the experimentally observed synaptic dynamics can be interpreted as supporting evidence for the theory that the mammalian brain implements TD learning. In the model there is a strong interaction between changes on the behavioral and on the synaptic level; modifications of synaptic strengths have an impact on the agent's choice, whereas the agent's choice determines the change in synaptic efficacy. This work can therefore be seen as a step towards a better understanding between synaptic plasticity and system-level learning taking place on completely different temporal and spatial scales. For other examples of modeling studies which similarly aim to bridge the considerable distance between these two levels of description, see [24], [33], [34], [60]–[62].

We developed our model by combining a top-down with a bottom-up approach, which we think is the best approach to try and understand multi-scale dynamics. A purely top-down approach is under-constrained. Developing a model solely to provide a specific function can in principle result in many different architectures with no guarantee of biological plausibility. Conversely, a purely bottom-up approach starting from experimentally observed properties of neurons and synapses tends to generate models that are too complex to be understood. Moreover, it is very unlikely that a model developed in this way will spontaneously exhibit a complex functionality on the behavioral level. By combining the two approaches we can develop models that are biologically plausible, account for multiple experimental findings and yet are still simple enough to yield insights into the mechanisms of information processing in the brain. In the following, we will discuss the significance of our results and the limits, predictions and future directions of this study.

### Learning performance on the grid-world task

The learning speed and performance of the neuronal network on the grid-world task with sparse positive reward are comparable to that of a discrete-time actor-critic TD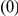 learning implementation. In some respects this result is not surprising, as the plasticity dynamics were designed to fulfill the main properties of TD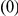 learning: value function and policy updates are proportional to the TD error and modifications are applied only with respect to the most recently exited state and the most recently chosen action. However, the dopaminergic signal does not perfectly reproduce the characteristics of the algorithmic TD error signal. The amplitude of the phasic activity is a nonlinear function of the difference in value between two states, and the dynamic range for negative errors is small. Moreover, synapses are not only updated due the presence of an error signal, but also due to small fluctuations of the dopaminergic firing rate around the baseline firing rate. Finally, the timing condition given by the product of the pre-synaptic efficacy and the pre-synaptic activity trace is not as strict as that defined by the discrete-time updates. Consequently, synapses undergo minor changes outside of the desired period of sensitivity.

The learning speed of our model is better than that exhibited by an earlier proposed TD learning model on the same task [34]. The major difference between the two models is that in the previously proposed model, each synapse calculates its own approximation of the TD error based on a comparison of two post-synaptic activity traces with different time constants, whereas in the model presented here the TD error is represented as a population signal available to all synapses. This suggests that a population signal is a more reliable method for the brain to represent reward information.

Although the grid-world task resembles a navigational task, it has more in common with an abstract association task such as learning associations between pairs of words, as the neuronal agent has no ability to exploit information about the underlying grid-world structure. This is also the reason why the agent requires many more trials to converge to a good performance than a rat requires to reliably find a hidden platform in a watermaze experiment [63]. Considerably faster convergence times have been demonstrated by reinforcement learning methods if the underlying structure of the environment is incorporated into the algorithm, for example by assuming overlapping state representations [29], [39].

In our model, all states are initialized to the same value, reflecting the assumption that the agent knows nothing about the proximity of the states to the reward position at the outset. After the task has been learnt, a gradient is developed with higher values around the reward state. Clearly, it will take the agent longer to re-learn a new reward position far away from the previous one than it took to learn the original position, as the gradient has to be unlearnt. In contrast, rats re-learn a modified task much faster than they learnt the original task [63]. Faster re-learning has been demonstrated in a non-spiking actor-critic model when the agent learns an abstract internal state representation in addition to the value function and policy [39]. Interestingly, it has been shown that mice with suppressed adult neurogenesis also show highly specific learning deficits, especially in re-learning, which demonstrates the importance of newly generated neurons [64]. In future work we will extend our model to investigate the relationship between neurogenesis, internal state representation and the speed of re-learning a modified task.

We have chosen the grid-world task to study the learning behavior of the proposed network model, as the complexity of the task makes it an adequate test case for TD learning algorithms. However, in experimental set-ups the role of dopamine in reward learning is typically studied in conditioning tasks, where a single stimulus is followed by a delayed reward. In order to test our network in such tasks requires an input representation different from the discrete state representation chosen in our model. Typically, in TD learning models such a stimulus is represented as a complete serial compound [2], [4]. Here, the stimulus is represented by a vector, where the 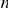
th entry represents the stimulus 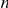 time steps into the future. Such a representation requires the system to know the number of time steps between the stimulus presentation and the reward delivery. A biologically more plausible representation of stimuli has recently been presented in [65]. Here the complete serial compound is replaced by a series of internal overlapping microstimuli. It has been demonstrated that such a representation results in a TD error in good agreement with most experimental findings on the dopaminergic activity during conditioning experiments [65]. It remains to be investigated in how far such a state representation can be adapted to spiking neuronal networks.

### Learning performance on the cliff-walk task

Due to its low baseline level, the dopaminergic firing rate has a much smaller dynamic range available for the representation of negative errors than for positive errors. In the literature two main possibilities to represent negative TD errors have been discussed. One possibility is that negative errors are represented by a different neuromodulator such as serotonin [66]. Another possibility is that negative errors are encoded in the duration of the phasic pauses in the dopamine neurons [46], suggesting that one neurotransmitter is enough to encode negative as well as positive errors. The latter hypothesis is supported in a modeling study demonstrating that dopamine is able to encode the full range of TD errors when the external stimuli are represented by a series of internal microstimuli [65]. Our study on the cliff-walk task with purely negative rewards reveals an additional problem to that of representing negative TD errors: due to their inherent lower bound the cortico-striatal synapses are limited in their ability to store estimates of future negative rewards.

A possible hypothesis that would also allow learning to be driven by purely negative rewards is that the absolute values of the estimates of future negative rewards are stored in different synaptic structures from those storing estimates of future positive rewards. This hypothesis is in line with experimental studies in rats and humans showing a functional segregation within the striatum, with anterior regions responding more strongly to positive rewards and posterior regions to negative rewards [67]–[69]. An analogous segregation has also been reported between the amygdala and the ventral striatum, with the former responding only to losses and the latter to gains [70]. Our results support the hypothesis that prediction errors with respect to negative rewards are represented by a different neuromodulator and possibly a different anatomical system, rather than the duration of the phasic pauses in the dopamine neurons. On the other hand, they are compatible with a hybrid strategy in which the brain uses both mechanisms: a neuromodulator other than dopamine to encode negative errors due to punishment, and the phasic pauses in the dopaminergic firing rate to represent disappointment about an omitted reward. These hypotheses could be differentiated by tests on patients with Parkinson's disease or on animal Parkinson's models. In either case, we predict that learning is less impaired when driven by external negative rewards than by positive ones. The extent of the learning impairment in tasks where reward omission plays an important role will further discriminate whether the brain relies on dopamine or some other system to signal such events.

### Model architecture

We investigated to what extent a top-down derived plasticity model dependent on the dynamics of a dopaminergic signal with realistic firing rates is able to implement the TD(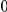) algorithm. For this purpose we assumed a very simplified model of the basal ganglia adapted from [18]. The key feature for our model is that the critic module dynamically generates a realistic error signal in response to the development of the value function encoded in the cortico-striatal synapses and the chosen action, rather than artificially generating a perfect error signal outside of the network. The mechanism by which the dopaminergic error signal is generated by the basal ganglia is as yet unknown, and answering this question is outside the scope of this manuscript. The architecture of the critic module assumed in our model uses an indirect and a delayed direct pathway from the striatum to the dopamine neurons to produce an error signal with activity and temporal features similar to those experimentally. We implement the slowness of the direct pathway by a long synaptic delay; a more biologically realistic realization could be 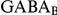 receptors, which are known to mediate slow inhibitory processes. Indeed, high densities of 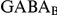 receptors have been found in the substantia nigra [71]. However, there are contradictory findings on whether the inhibitory response of the dopamine neurons is mediated by 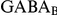. Whereas in vitro inhibitory responses in midbrain dopamine neurons can be mediated by 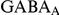 and 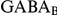 [72], [73], in vivo studies in rats have reported that the synaptic connections between the neurons in the striatum and dopamine neurons in the substantia nigra act predominantly or exclusively via the 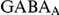 receptors [74], [75]. However, a recent in vivo study in mice found that after stimulation of the striatum, dopamine neurons in the substantia nigra show a long lasting inhibition mediated by 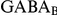 receptors absent in rats [76].

Future experimental studies may reveal whether the dopaminergic signal is indeed generated by a fast indirect path and a slow direct pathway, or by some other mechanism [22]. Some alternative actor-critic models of the basal ganglia are discussed in [23]. Most of the alternative models make assumptions that are experimentally not well supported. For example, several models assume a direct excitatory pathway and an indirect inhibitory pathway between the striatum and the dopamine neurons [4], [19]–[21], [77], whereas in reality the situation is reversed [23]. A model that basically resembles that proposed by Houk et al. [18] but implements several known anatomical structures more accurately than any other model was presented in [78]. However, this model relies on three-factor synaptic plasticity rules for striato-nigral connections, for which there is no experimental evidence. This assumption is also made in [79]. Some of the alternative models also posit a divergent architecture, in which the input arises from two different sources [79], [80]. Due to the different timing properties along the two divergent pathways, the model proposed in [80] is able to reproduce most of the known experimental data. However, where parallel reciprocal architectures such as those proposed in [4], [18]–[21], [77] can be directly related to TD learning, the same is not true for divergent or non-reciprocal architecture [23]. The generation mechanism may also depend on pathways within the basal ganglia that have so far been neglected in modeling studies. For example, input from the lateral habenula to the dopamine neurons has recently been shown to be an important source of negative inputs to the dopamine neurons [81].

The focus of our work is action learning rather than action selection. Consequently, we have kept the actor module as simple as possible. One disadvantage of this choice is its vulnerability: if one actor neuron dies, the action that is represented by that neuron can never be chosen again. Furthermore, the inhibition of the actor neurons after an action has been chosen is applied externally rather than arising naturally through the network dynamics. Candidate action selection mechanisms that would overcome these limitations include attractor networks [82] and competing synfire chains [83]–[85]. Moreover, we have not related the action module to any specific brain region. Imaging experiments have found that the activity in the ventral striatum is correlated with the TD error during a prediction and action selection task, whereas the activity in the dorsal striatum is correlated with the TD error only during the action selection task [10], [86]. In the context of the actor-critic architecture, this finding implies that the ventral striatum performs the role of the critic and the dorsal striatum performs the role of the actor. Detailed models have been developed that relate the problem of action selection to loops through the basal ganglia [41], [42] and also loops through the cerebellum and the cerebral cortex [87], [88]. An overview of different basal ganglia models that especially focuses on the action selection problem can be found in [89].

### Dependence on model size

The error signal in our model is encoded in the difference between the dopaminergic population firing rate from its baseline level. The learning behavior of the model therefore depends on the number of dopamine neurons generating the population signal and the noise of this signal. As learning is driven by fluctuations in the dopaminergic firing rate from the baseline level, a noisier signal will drive the learning process less efficiently. A thorough investigation of the effects of model size and noise is outside the scope of this article, however, it is possible to extrapolate some of these effects from the dynamics of our model.

We have shown that even as few as 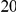 dopamine neurons generate a signal that is sufficiently reliable to learn the tasks investigated here. Increasing the number of neurons, assuming the synaptic baseline reference is correspondingly increased, would have the effect of reducing the noise in the dopamine signal. However, as the neuronal network model already performs as well as the discrete-time algorithm, no performance improvement can be expected. Conversely, decreasing the number of dopaminergic neurons reduces both the amplitude of the phasic signal and the baseline activity and makes the remaining signal noisier and less reliable.

Even assuming a perfectly reliable signal, the dynamics developed in our model are such that if the synaptic baseline reference is not reduced accordingly, the lower baseline activity appears in the synaptic plasticity dynamics as a permanent negative error signal. This depresses the synaptic weights that encode the value function and policy until they reach their minimum values. At this point the agent can no longer distinguish between states with respect to their reward proximity and has no preference for any action over any other action. Moreover, decreasing the synaptic weights that encode the policy slows the responses of the actor neurons and therefore leads to slower decision processes. Analogous behavior has been observed in patients with Parkinson's disease, which is characterized by a gradual loss in the number of dopamine neurons, who show movement as well as cognitive deficits [90].

The dynamics of our model predicts that increasing background dopamine concentration after a gradual loss in dopamine neurons maintains any existing memory of state values, as it will restore the amount of available dopamine to the baseline level used as a reference by the synapse. However, learning in new tasks is still impaired, as this is driven by fluctuations in the dopaminergic signal rather than its baseline level. The reduced remaining population of dopaminergic neurons necessarily produces smaller and noisier fluctuations than those generated by an intact population; consequently, they provide a less effective learning signal. This is an equivalent situation to reducing the size of the dopamine population and reducing the baseline reference value in the synapse accordingly. This prediction is consistent with the finding that even fully medicated Parkinson's patients exhibit deficits in a probabilistic classification task [91]. The dynamics of the critic module also predicts that the size of the striatal population should also be critical for the learning behavior, as it determines the amplitude of the phasic dopaminergic signal. This is in agreement with studies showing that a lesion of the dorsal striatum impairs the learning behavior of rats in stimulus-response learning [92].

### Synaptic plasticity dynamics realizing TD learning

The plasticity dynamics presented in Eq. (8) is in some degree similar to the plasticity dynamics derived in our previous investigation of a spiking neuronal network model capable of implementing actor-critic TD learning [34]. The two plasticity dynamics have in common that the dynamics is triggered by biologically plausible measures of the pre-synaptic activity and is dependent on a TD error signal. However, in our earlier model there is no dopaminergic error signal available; each synapse performs its own approximation of an TD error based on the difference in a rapid and a laggard post-synaptic activity trace. The aim was to develop a continuous-time plasticity mechanism that mapped the properties of the discrete-time TD learning algorithm as accurately as possible. Thus, the study can be seen as a proof of principle that a spiking neuronal network model can implement actor-critic TD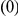 learning. On the basis of this, in our current study we focus on applying biological constraints to the range of possible plasticity dynamics by combining the previous top-down approach with a bottom-up approach.

The biological constraints entailed by our use of a dopaminergic error signal with realistic firing rates to represent the TD error lead to two major differences from the original plasticity mechanism developed in [34]. First, whereas the plasticity dynamics presented in the previous model belongs to the class of differential Hebbian learning rules modulated with a non-local constant reward signal, in the model presented here, the plasticity dynamics belongs to the class of neuromodulated, heterosynaptic plasticity. Second, whereas the earlier synaptic plasticity dynamics can be mapped exactly to the value function update of TD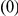 learning, the plasticity dynamics presented here corresponds to a slightly modified TD learning algorithm with self-adapting learning parameters.

Our finding that the learning parameters 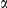 and 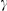 increase with the difference in successive cortico-striatal synaptic weights 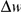 could be tested experimentally by fitting TD learning algorithms to behavioral data gathered from animals learning two versions of a task: one with large rewards and one with small rewards. As long as 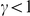, the task with larger rewards will develop greater differences in the estimation of future rewards of successive states than the task with smaller rewards. We therefore predict that the values of the learning parameters 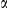 and 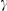 fitted to the former set of behavioral data will be greater those fitted to the latter set. Additionally, the values calculated by fitting 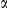 and 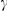 to different epochs in behavioral data gathered from an animal learning a given task should vary in a systematic fashion. At the very beginning, the animal presumably has no expectations about future rewards and thus estimates all states similarly. During the middle of the learning process, when the animal's performance is improving rapidly, large differences between the estimation of states can be expected. Finally, as the animal approaches its equilibrium performance, differences between the estimations of states should vary smoothly. We therefore predict that fitting 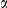 and 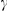 to data gathered from the beginning and end of the learning process will result in lower values than fitting the learning parameters to data gathered whilst the performance on a given learning task is improving rapidly.

### TD learning and the brain

Is actor-critic TD learning the correct model? This is outside the scope of the current manuscript, and perhaps out of our remit altogether - this kind of question can only be answered by analyzing behavioral, electrophysiological and anatomical data from carefully designed experiments. There is evidence on the behavioral american [93] as well as on the cellular level american [2], [9] that mammals implement TD learning strategies. TD learning has been successfully applied to model bee foraging in uncertain environments american [19], [94], human decision making american [4] and rat navigation american [39], but it is unlikely to be the only learning strategy used by the brain [95]. In line with previous studies [10], [18], [20], we have focused on TD learning with the actor-critic architecture instead of other TD learning methods, such as SARSA or Q-learning [1]. However, recent experimental findings also support the interpretation that mammals implement TD learning methods based on action values [17] or an actor-director model [14]. Further research is needed, especially on the theoretical side, in order to understand if these models are compatible with spiking neuronal networks.

We have focused on the simplest TD learning algorithm: TD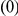. However, it is likely that the mammalian brain uses more advanced TD learning strategies. TD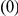 learning is efficient as long as the number of possible states and actions are restricted to a small to moderate number. To address problems with a large number of states and possible actions, TD learning methods that generalize from a small number of observed states and chosen actions are needed (see [1]). Furthermore, it has been demonstrated that classical TD learning schemes cannot account for behavioral data involving motivation. Modified TD algorithms can explain these data, either by explicitly including a motivational term [96] or by ‘average-reward TD-learning’, where an average reward acts as a baseline [97].

Here, we have interpreted the phasic dopaminergic signal in the light of TD learning. However, the literature presents a much broader picture of the functional role of the dopaminergic activity. It has been found that only a small subgroup of dopamine neurons show a response consistent with the TD error hypothesis; a much broader group responds with an increase in activity to positive as well as negative reward related signals inconsistent with the hypothesis [98]. There is also evidence that dopamine is involved with signalling ‘desire’ for a reward rather than the reward itself [99], [100]. Furthermore, the phasic dopaminergic signal responds to a much larger category of events than just to reward related events, including aversive, high intensity or novel stimuli [101]. Alternative interpretations of the phasic signal include the theory that it acts more like a switch than a reward signal, triggering learning at the right point in time [102], [103], or that it promotes the discovery of new actions and learning of new action-outcome associations, independent of the economic value of the action [5]. Given the diversity of dopaminergic responses and considering the fact that midbrain dopamine neurons project to many different brain areas, such as the striatum, the orbifrontal cortex and the amygdala [3], it is also likely that different interpretations are simultaneously valid; the information encoded in the phasic signal being combined with local information in specific areas of the brain to realize a variety of functions.

## Methods

### Neuronal network simulations

We investigated our model using numerical simulations. We implemented the model in the simulator NEST [104] and performed the simulations in parallel on two nodes of a cluster of 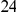 SUN X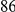 machines, each with two 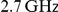 AMD Opteron 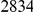 quad core processors running Ubuntu Linux. The dopamine modulated plasticity dynamics Eq. (8) and Eq. (13) are implemented employing the distributed simulation framework presented in [105].

All neurons in the network are modeled as current-based integrate-and-fire neurons. The dynamics of the membrane potential for each neuron is given by:
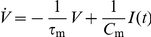
where 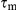 is the time constant, 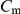 the capacity of the membrane and 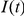 the input current to the neurons [106]. When 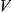 reaches a threshold 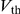, a spike is emitted. The membrane potential is subsequently clamped to 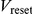 for the duration of an absolute refractory period 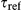. The synaptic current due to an incoming spike is represented as an 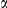-function
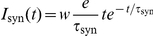
where 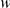 is the peak amplitude and 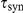 the rise time. The neuronal parameters are specified in the following section.

### Model description and parameter specification

**Figure 12:**
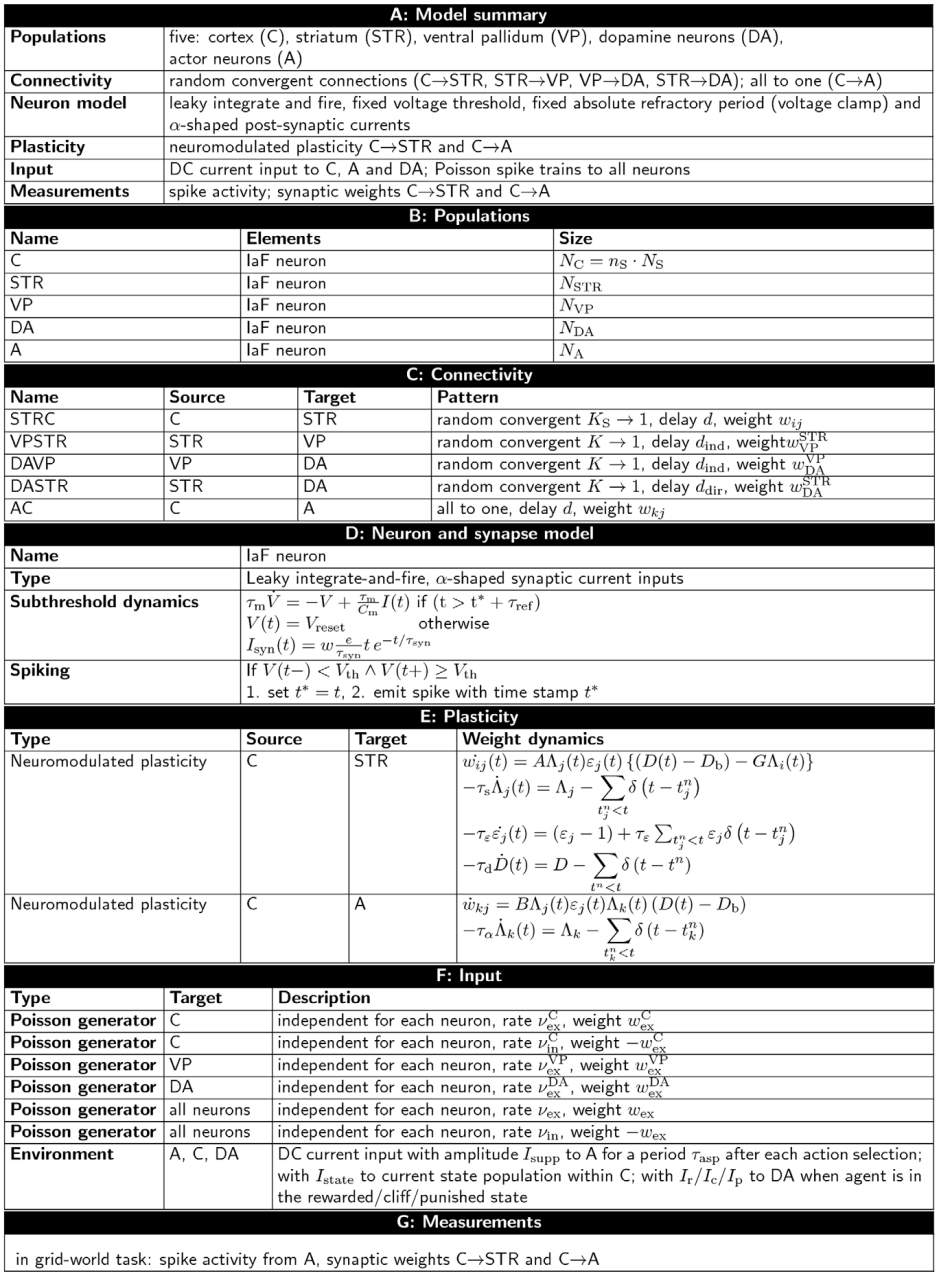
Model description after [**107**].

**Figure 13:**
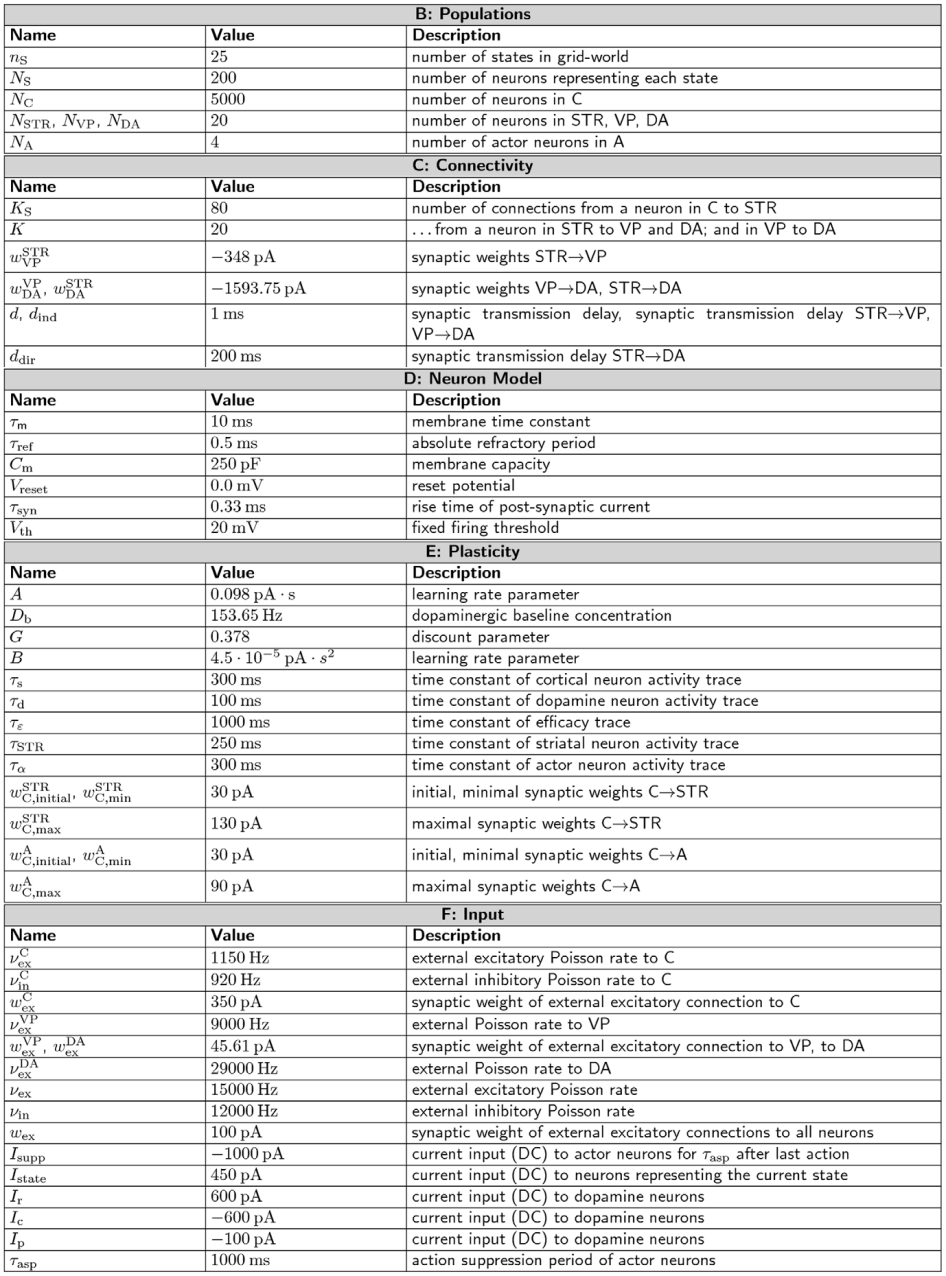
Parameter specification. The categories refer to the model description in Fig. 12.

The details of the model are summarized in Fig. 12 using the scheme developed by [107]. The parameters used in the numerical simulations are specified in Fig. 13.

## Supporting Information

Text S1 Conditions for a constant dopaminergic baseline firing rate.
(0.14 MB PDF)

Text S2 Mapping parameters.
(0.19 MB PDF)

## References

[1] Sutton RS, Barto AG (1998). Reinforcement Learning: An Introduction. Adaptive Computation and Machine Learning.

[2] Schultz W, Dayan P, Montague PR (1997). A neural substrate of prediction and reward.. Science.

[3] Schultz W (2002). Getting formal with dopamine and reward.. Neuron.

[4] Montague PR, Dayan P, Sejowski TJ (1996). A framework for mesencephalic dopamine systems based on predictive Hebbian learning.. J Neurosci.

[5] Redgrave P, Gurney K (2006). The short-latency dopamine signal: a role in discovering novel actions?. Nat Rev Neurosci.

[6] Pessiglione M, Seymour B, Flandin G, Dolan R, Frith C (2006). Dopamine-dependent prediction errors underpin reward-seeking behaviour in humans.. Nature.

[7] Reynolds JNJ, Hyland BI, Wickens JR (2001). A cellular mechanism of reward-related learning.. Nature.

[8] Pawlak V, Kerr JN (2008). Dopamine receptor activation is required for corticostriatal spike-timing-dependent plasticity.. J Neurosci.

[9] Reynolds JN, Wickens JR (2002). Dopamine-dependent plasticity of corticostriatal synapses.. Neural Netw.

[10] O'Doherty J, Dayan P, Schultz J, Deichmann R, Friston K (2004). Dissociable roles of ventral and dorsal striatum in instrumental conditioning.. Science.

[11] Witten IH (1977). An adaptive optimal controller for discrete-time markov environments.. Information and Control.

[12] Barto A, Sutton RS, Anderson CW (1983). Neuronlike adaptive elements that can solve difficult learning control problems.. IEEE Trans Syst Man Cybern.

[13] Morris G, Nevet A, Arkadir D, Vaadia E, Bergman H (2006). Midbrain dopamine neurons encode decisions for future action.. Nat Neurosci.

[14] Attalah HE, Lopez-Paniagua D, Rudy JW, O'Reilly RC (2007). Separate neural substrates for skill-learning and performance in the ventral and dorsal striatum.. Nat Neurosci.

[15] Fiorillo CD, Tobler PN, Schultz W (2003). Discrete coding of reward probability and uncertainty by dopamine neurons.. Science.

[16] Tobler PN, Fiorillo CD, Schultz W (2005). Adaptive coding of reward value by dopamine neurons.. Science.

[17] Morris G, Arkadir D, Nevet A, Vaadia E, Bergman H (2004). Coincident but distinct messages of midbrain dopamine and striatal tonically active neurons.. Neuron.

[18] Houk JC, Adams JL, Barto AG (1995). A model of how the basal ganglia generate and use neural signals that predict reinforcement.

[19] Montague P, Dayan P, Person C, Sejnowski T (1995). Bee foraging in uncertain environments using predictive Hebbian learning.. Nature.

[20] Suri R, Schultz W (1999). A neural network model with dopamine-like reinforcement signal that learns a spatial delayed reponse task.. Neuroscience.

[21] Suri RE, Schultz W (2001). Temporal difference model reproduces anticipatory neural activity.. Neural Comput.

[22] Joel D, Niv J, Ruppin E (2002). Actor-critic models of the basal ganglia: new anatomical and computational perspectives.. Neural Netw.

[23] Wörgötter F, Porr B (2005). Temporal sequence learning, prediction, and control: A review of different models and their relation to biological mechanisms.. Neural Comput.

[24] Seung HS (2003). Learning spiking neural networks by reinforcement of stochastic synaptic transmission.. Neuron.

[25] Xie X, Seung HS (2004). Learning in neural networks by reinforcement of irregular spiking.. Phys Rev E.

[26] Baras D, Meir R (2007). Reinforcement learning, spike-time-dependent plasticity, and the BCM rule.. Neural Comput.

[27] Florian RV (2007). Reinforcement learning through modulation of spike-timing-dependent synaptic plasticity.. Neural Comput.

[28] Legenstein R, Pecevski D, Maass W (2008). A learning theory for reward-modulated spike-timing-dependent plasticity with application to biofeedback.. PLoS Comput Biol.

[29] Vasilaki E, Frémaux N, Urbanczik R, Senn W, Gerstner W (2009). Spike-based reinforcement learning in continuous state and action space: When policy gradient methods fail.. PLoS Comput Biol.

[30] Frémaux N, Sprekeler H, Gerstner W (2010). Functional requirements for reward-modulated spike-timing-dependent plasticity.. J Neurosci.

[31] Rao RPN, Sejnowski TJ (2001). Spike-timing-dependent Hebbian plasticity as temporal difference learning.. Neural Comput.

[32] Farries MA, Fairhall AL (2007). Reinforcement learning with modulated spike timing-dependent synaptic plasticity.. J Neurophysiol.

[33] Izhikevich EM (2007). Solving the distal reward problem through linkage of STDP and dopamine signaling.. Cereb Cortex.

[34] Potjans W, Morrison A, Diesmann M (2009). A spiking neural network model of an actor-critic learning agent.. Neural Comput.

[35] Dennett DC (1998). Brainchildren: Essays on Designing Minds.

[36] Barto AG, Houk JC, Davis J, Beiser D (1995). Adaptive critic and the basal ganglia.. Models of Information Processing in the Basal Ganglia.

[37] Dayan P (1992). The convergence of td(λ) for general λ.. Mach Learn.

[38] Dayan P, Sejnowski T (1994). Td(λ) converges with probability 1.. Mach Learn.

[39] Foster DJ, Morris RGM, Dayan P (2000). A model of hippocampally dependent navigation, using the temporal difference learning rule.. Hippocampus.

[40] VanRullen R, Guyonneau R, Thorpe SJ (2005). Spike times make sense.. Trends Neurosci.

[41] Gurney K, Prescott TJ, Redgrave P (2001). A computational model of action selection in the basal ganglia. i. a new functional anatomy.. Biol Cybern.

[42] Humphries MD, Stewart RD, Gurney KN (2006). A physiologically plausible model of action selection and oscillatory activity in the basal ganglia.. J Neurosci.

[43] Prinz AA, Bucher D, Marder E (2004). Similar network activity from disparate circuit parameters.. Nat Neurosci.

[44] Dai M, Tepper JM (1998). Do silent dopaminergic neurons exist in rat substantia nigra in vivo?. Neuroscience.

[45] Hyland BI, Reynolds JNJ, Hay J, Perk CG, Miller R (2002). Firing modes of midbrain dopamine cells in the freely moving rat.. Neuroscience.

[46] Bayer HM, Lau B, Glimcher PW (2007). Statistics of midbrain dopamine neuron spike trains in the awake primate.. J Neurophysiol.

[47] Ljungberg T, Apicella P, Schultz W (1992). Responses of monkey dopamine neurons during learning of behavioral reactions.. J Neurophysiol.

[48] Schultz W (1998). Predictive reward signal of dopamine neurons.. J Neurophysiol.

[49] Helias M, Deger M, Rotter S, Diesmann M (2010). Instantaneous non-linear processing by pulse-coupled threshold units.. PLoS Comput Biol.

[50] Froemke RC, Dan Y (2002). Spike-timing-dependent synaptic modification induced by natural spike trains.. Nature.

[51] Garris PA, Ciolkowski EL, Pastore P, Wightman RM (1994). Efflux of dopamine from the synaptic cleft in the nucleus accumbens of the rat brain.. J Neurosci.

[52] Montague PR, McClure SM, Baldwin P, Phillips PE, Budygin EA (2004). Dynamic gain control of dopamine delivery in freely moving animals.. J Neurosci.

[53] Soltani A, Lee D, Wang XJ (2006). Neural mechanism for stochastic behavior during a competitive game.. Neural Netw.

[54] Schweighofer N, Doya K (2003). Meta-learning in reinforcement learning.. Neural Comput.

[55] Friston KJ, Tononi G, Reeke GN, Sporns O, Edelman GM (1994). Value-dependent selection in the brain: Simulation in a synthetic neural model.. Neuroscience.

[56] Calabresi P, Fedele E, Pisani A, Fontana G, Mercuri N (1995). Transmitter release associated with long-term synaptic depression in rat corticostriatal slices.. Eur J Neurosci.

[57] Wickens J (1993). A Theory of the Striatum..

[58] Pawlak V, Wickens JR, Kirkwood A, Kerr JND (2010). Timing is not everything: neuromodulation opens the STDP gate.. Front Syn Neurosci.

[59] Nakano T, Doi T, Yoshimoto J, Doya K (2010). A kinetic model of dopamine- and calcium-dependent striatal synaptic plasticity.. PLoS Comput Biol.

[60] Loewenstein Y, Seung HS (2006). Operant matching is a generic outcome of synaptic plasticity based on the covariance between reward and neural activity.. Proc Natl Acad Sci USA.

[61] Fusi S, Asaad WF, Miller EK, Wang XJ (2007). A neural circuit model of flexible sensorimotor mapping: learning and forgetting on multiple timescales.. Neuron.

[62] Soltani A, Wang XJ (2010). Synaptic computation underlying probabilistic inference.. Nat Neurosci.

[63] Steele RJ, Morris RGMM (1999). Delay-dependent impairment of a matching-to-place task with chronic and intrahippocampal infusion of the nmda-antagonist d-ap5.. Hippocampus.

[64] Garthe A, Behr J, Kempermann G (2009). Adult-generated hippocampal neurons allow the flexible use of spatially precise learning strategies.. PLoS ONE.

[65] Ludvig EA, Sutton RS, Kehoe EJ (2008). Stimulus representation and the timing of reward-prediction errors in models of the dopamine system.. Neural Comput.

[66] Daw ND, Kakade S, Dayan P (2002). Opponent interactions between serotonin and dopamine.. Neural Networks.

[67] Reynolds SM, Berridge KC (2001). Fear and feeding in the nucleus accumbens shell: Rostrocaudal segregation of gaba-elicited defensive behavior versus eating behavior.. J Neurosci.

[68] Reynolds SM, Berridge KC (2002). Positive and negative motivation in nucleus accumbens shell: Bivalent rostrocaudal gradients for gaba-elicited eating, taste “liking”/“disliking” reactions, place preference/avoidance, and fear.. J Neurosci.

[69] Seymour B, Daw N, Dayan P, Singer T, Dolan R (2007). Differential encoding of losses and gains in the human striatum.. J Neurosci.

[70] Yacubian J, Gläscher J, Schroeder K, Sommer T, Braus DF (2006). Dissociable systems for gain- and loss-related value predictions and errors of prediction in the human brain.. J Neurosci.

[71] Bowery N, Hudson A, Price G (1987). Gabaa andgabab receptor site distribution in the rat central nervous system.. Neuroscience.

[72] Häusser MA, Yung WH (1994). Inhibitory synaptic potentials in guinea-pig substantia nigra dopamine neurones in vitro.. J Physiol.

[73] Sugita S, Johnson SW, North RA (1992). Synaptic inputs to gabaa and gabab receptors originate from discrete afferent neurons.. Neurosci Lett.

[74] Tepper JM, Martin L, Anderson DR (1995). Gabaa receptor-mediated inhibition of rat substantia nigra dopaminergic neurons by pars reticulata projection neurons.. J Neurosci.

[75] Paladini CA, Celada P, Tepper JM (1999). Striatal, pallidal, and pars reticulata evoked inhibition of nigrostriatal dopaminergic neurons is mediated by gabaa receptors in vivo.. Neuroscience.

[76] Brazhnik E, Shah F, Tepper JM (2008). Gabaergic afferents activate both gabaa and gabab receptors in mouse substantia nigra dopaminergic neurons in vivo.. J Neurosci.

[77] Suri RE, Bargas J, Arbib MA (2001). Modeling functions of striatal dopamine modulation in learning and planning.. Neuroscience.

[78] Berns GS, Sejnowski TJ (1998). A computational model of how the basal ganglia produce sequences.. J Cogn Neurosci.

[79] Contreras-Vidal JL, Schultz W (1999). A predictive reinforcement model of dopamine neurons for learning approach behavior.. J Comput Neurosci.

[80] Brown J, Bullock D, Grossberg S (1999). How the basal ganglia use parallel excitatory and inhibitory learning pathways to selectively respond to unexpected rewarding cues.. J Neurosci.

[81] Matsumoto M, Hikosaka O (2007). Lateral habenula as a source of negative reward signals in dopamine neurons.. Nature.

[82] Hopfield JJ (1982). Neural networks and physical systems with emergent collective computational abilities.. Proc Natl Acad Sci USA.

[83] Jin DZ (2009). Generating variable birdsong syllable sequences with branching chain networks in avian premotor nucleus HVC.. Phys Rev E.

[84] Hanuschkin A, Herrmann JM, Morrison A, Diesmann M (2010). Compositionality of arm movements can be realized by propagating synchrony.. J Comput Neurosci.

[85] Schrader S, Diesmann M, Morrison A (2010). A compositionality machine realized by a hierarchic architecture of synfire chains.. Front Comput Neurosci.

[86] Seymour B, O'Doherty J, Dayan P, Koltzenburg M, Jones A (2004). Temporal difference models describe higher-order learning in humans.. Nature.

[87] Houk JC, Wise SP (1995). Distributed modular architectures linking basal ganglia, cerebellum, and cerebral cortex: Their role in planning and controlling action.. Cereb Cortex.

[88] Houk JC (2005). Agents of the mind.. Biol Cybern.

[89] Houk JC (2007). Models of basal ganglia.. Scholarpedia.

[90] Sethi KD (2002). Clinical aspects of parkinson disease.. Curr Opin Neurol.

[91] Knowlton BJ, Mangels JA, Squire LR (1996). A neostriatal habit learning system in humans.. Science.

[92] McDonald RJ, White NM (1993). A triple dissociation of memory systems: hippocampus, amygdala, and dorsal striatum.. Behav Neurosci.

[93] Sutton RS, Barto AG, Gabriel M, Moore J (1990). Time-derivative models of pavlovian reinforcement.. Learning and Computational Neuroscience.

[94] Niv Y, Joel D, Meilijson I, Ruppin E (2002). Evolution of reinforcement learning in uncertain environments: A simple explanation for complex foraging behaviors.. Adapt Behav.

[95] Doya K (2000). Complementary roles of basal ganglia and cerebellum in learning and motor control.. Curr Opin Neurol.

[96] La Camera G, Richmond BJ (2008). Modeling the violation of reward maximization and invariance in reinforcement schedules.. PLoS Comput Biol.

[97] Dayan P (2009). Prospective and retrospective temporal difference learning.. Network Comput Neural Syst.

[98] Matsumoto M, Hikosaka O (2009). Two types of dopamine neuron distinctly convey positive and negative motivational signals.. Nature.

[99] Arias-Carrion O, Pöppel E (2007). Dopamine, learning, and reward-seeking behavior.. Acta Neurobiol Exp (Wars).

[100] Pecina S, Cagniard B, Berridge KC, Aldridge JW, Zhuang X (2003). Hyperdopaminergic mutant mice have higher “wanting” but not “liking” for sweet rewards.. J Neurosci.

[101] Horvitz JC (2000). Mesolimbocortical and nigrostriatal dopamine responses to salient non-reward events.. Neuroscience.

[102] Redgrave P, Prescott TJ, Gurney K (1999). Is the short-latency dopamine response too short to signal reward error?. Trends Neurosci.

[103] Porr B, Wörgötter F (2007). Learning with relevance: Using a third factor to stabilise hebbian learning.. Neural Comput.

[104] Gewaltig MO, Diesmann M (2007). NEST (NEural Simulation Tool).. Scholarpedia.

[105] Potjans W, Morrison A, Diesmann M (2010). Enabling functional neural circuit simulations with distributed computing of neuromodulated plasticity.. Front Comput Neurosci.

[106] Tuckwell HC (1988). Introduction to Theoretical Neurobiology, volume 1.

[107] Nordlie E, Gewaltig MO, Plesser HE (2009). Towards reproducible descriptions of neuronal network models.. PLoS Comput Biol.

