# Supplementary material for: An Imperfect Dopaminergic Error Signal Can Drive Temporal-Difference Learning
Source: PLoS Comput Biol. 2011 May 12;7(5):e1001133. doi: 10.1371/journal.pcbi.1001133 (PMC3093351; doi:10.1371/journal.pcbi.1001133)
Supplement: Text S1 — Conditions for a constant dopaminergic baseline firing rate. (0.14 MB PDF) [file pcbi.1001133.s001.pdf]

Supplementary Material S1: Conditions for a Constant Dopaminergic Baseline Firing Rate

It has been found that midbrain dopamine neurons react to a reward or reward predicting stimulus. As long as nothing unpredictable happens, the neurons fire with a constant baseline activity [1]. To incorporate this finding into our model, we need to find conditions such that the dopaminergic neurons fire with a constant baseline activity, independent of the value of the agent's current state, except during phasic activation following a state transition. Here, we derive two relationships that enable the network to fulfill this requirement: firstly between the number of neurons in the ventral pallidum N_{VP} and the number of neurons in the striatum N_{STR}, secondly between the weights of the synapses that connect the striatum to the dopaminergic neurons $w_{\mathrm{DA}}^{\mathrm{STR}}$ and those that connect the ventral pallidum to the dopaminergic neurons $w_{\mathrm{DA}}^{\mathrm{VP}}$.

The firing rate λ of an integrate-and-fire neuron can be calculated from the first passage time of the membrane potential across the threshold (see [2] for a review). For leaky integrate-and-fire neuron models, provided the distribution of the free membrane potential is sufficiently close to a Gaussian, the mean μ and the variance σ^2 of this distribution determine the first passage time and therefore also the firing rate:

$$\lambda = f\left(\mu, \sigma^2\right)$$

If an agent is stationary in a state with no associated external reward, each dopamine neuron integrates inputs from three sources: N_{STR} striatal neurons, each firing at λ_{STR} with mean synaptic weight $w_{\mathrm{DA}}^{\mathrm{STR}}$, N_{VP} neurons of the ventral pallidum, each firing at λ_{VP} with mean synaptic weight $w_{\mathrm{DA}}^{\mathrm{VP}}$, and the background noise. This last term is made up of an excitatory and an inhibitory Poissonian input with firing rates $\lambda_{\mathrm{bg,E}} = \lambda_{\mathrm{bg,I}} = \lambda_{\mathrm{bg}}$ and synaptic strengths $w_{\mathrm{bg,E}} = -w_{\mathrm{bg,I}}$. Under the assumption that all input spike trains are uncorrelated, stationary Poisson processes, the mean membrane potential μ and the variance σ^2 can be calculated according to shot noise theory [3]:

$$\mu = N_{\mathrm{STR}}\lambda_{\mathrm{STR}} \int_{-\infty}^{\infty} h\left(w_{\mathrm{DA}}^{\mathrm{STR}}, t\right) dt + N_{\mathrm{VP}}\lambda_{\mathrm{VP}} \int_{-\infty}^{\infty} h\left(w_{\mathrm{DA}}^{\mathrm{VP}}, t\right) dt$$

$$\sigma^2 = N_{\mathrm{STR}}\lambda_{\mathrm{STR}} \int_{-\infty}^{\infty} h^2\left(w_{\mathrm{DA}}^{\mathrm{STR}}, t\right) dt + N_{\mathrm{VP}}\lambda_{\mathrm{VP}} \int_{-\infty}^{\infty} h^2\left(w_{\mathrm{DA}}^{\mathrm{VP}}, t\right) dt + \lambda_{\mathrm{bg,E}} \int_{-\infty}^{\infty} h^2\left(w_{\mathrm{bg,E}}, t\right) dt + \lambda_{\mathrm{bg,I}} \int_{-\infty}^{\infty} h^2\left(w_{\mathrm{bg,I}}, t\right) dt,$$

where $h(w,t)$ is the post-synaptic potential. For α-shaped postsynaptic currents we have:

$$h(w,t)=\begin{cases}\frac{ew}{\tau_\alpha C}\left(\frac{1}{\tau_{\mathsf{m}}}-\frac{1}{\tau_\alpha}\right)^{-2}\left(\left[\frac{1}{\tau_{\mathsf{m}}}-\frac{1}{\tau_\alpha}\right]te^{-t/\tau_\alpha}-e^{-t/\tau_\alpha}+e^{-t/\tau_{\mathsf{m}}}\right) & t\geq 0\\ 0 & t<0\end{cases}$$

Therefore, the mean membrane potential and variance of a dopaminergic neuron are given by:

$$\mu=\left(N_{\mathrm{STR}}\lambda_{\mathrm{STR}}w_{\mathrm{DA}}^{\mathrm{STR}}+N_{\mathrm{VP}}\lambda_{\mathrm{VP}}w_{\mathrm{DA}}^{\mathrm{VP}}\right)F_1$$
$$\sigma^2=\left(N_{\mathrm{STR}}\lambda_{\mathrm{STR}}\left(w_{\mathrm{DA}}^{\mathrm{STR}}\right)^2+N_{\mathrm{VP}}\lambda_{\mathrm{VP}}\left(w_{\mathrm{DA}}^{\mathrm{VP}}\right)^2+2\lambda_{\mathrm{bg}}w_{\mathrm{bg}}^2\right)F_2$$

with

$$F_1 = \frac{1}{C}e\tau_{\mathsf{m}}\tau_\alpha$$
$$F_2 = \frac{1}{C^2}e^2\tau_{\mathsf{m}}^2\tau_\alpha^2\frac{(\tau_\alpha+2\tau_{\mathsf{m}})}{4\left(\tau_\alpha+\tau_{\mathsf{m}}\right)^2}\ .$$

We assume a linear relationship between the firing rates of the neurons in the ventral pallidum and striatal populations:

$$\lambda_{\mathrm{VP}} = -a\lambda_{\mathrm{STR}}+b$$

Therefore the mean membrane potential and variance of a dopaminergic neuron whilst the agent remains in an unrewarded state are given by:

$$\mu=\left(\left(w_{\mathrm{DA}}^{\mathrm{STR}}N_{\mathrm{STR}}-aw_{\mathrm{DA}}^{\mathrm{VP}}N_{\mathrm{VP}}\right)\lambda_{\mathrm{STR}}+bw_{\mathrm{DA}}^{\mathrm{VP}}N_{\mathrm{VP}}\right)F_1$$
$$\sigma^2=\left(\left(\left(w_{\mathrm{DA}}^{\mathrm{STR}}\right)^2N_{\mathrm{STR}}-a\left(w_{\mathrm{DA}}^{\mathrm{VP}}\right)^2N_{\mathrm{VP}}\right)\lambda_{\mathrm{STR}}+b\left(w_{\mathrm{DA}}^{\mathrm{VP}}\right)^2N_{\mathrm{VP}}+2\lambda_{\mathrm{bg}}\mathrm{w}_{\mathrm{bg}}^2\right)F_2$$

This results in the following conditions under which the dopaminergic firing rate is independent of the striatal firing rate λ_{STR} and hence of the value of the agent's current state:

$$w_{\mathrm{DA}}^{\mathrm{VP}} = \frac{1}{a}w_{\mathrm{DA}}^{\mathrm{STR}}\frac{N_{\mathrm{STR}}}{N_{\mathrm{VP}}}$$
$$\left(w_{\mathrm{DA}}^{\mathrm{VP}}\right)^2 = \frac{1}{a}\left(w_{\mathrm{DA}}^{\mathrm{STR}}\right)^2\frac{N_{\mathrm{STR}}}{N_{\mathrm{VP}}}$$

These conditions are fulfilled for

$$N_{\mathrm{STR}} = aN_{\mathrm{VP}}$$

and

$$w_{\mathrm{DA}}^{\mathrm{VP}} = w_{\mathrm{DA}}^{\mathrm{STR}}.$$

For the parameters used in our simulations, we have verified that the linear relationship between the firing rates of the neurons in the ventral pallidum and striatal populations holds with $a = 1$ resulting in a constant dopaminergic rate.

In such a network, when the agent moves from one state s_i to another state s_{i+1} the mean membrane potential and variance are given by:

$$\mu = \left(-w_{\mathrm{DA}}^{\mathrm{STR}} N_{\mathrm{STR}} \left[\lambda_{\mathrm{STR}}(s_{i+1}) - \lambda_{\mathrm{STR}}(s_i)\right] + b w_{\mathrm{DA}}^{\mathrm{VP}} N_{\mathrm{VP}}\right) F_1$$

$$\sigma^2 = \left(-\left(w_{\mathrm{DA}}^{\mathrm{STR}}\right)^2 N_{\mathrm{STR}} \left[\lambda_{\mathrm{STR}}(s_{i+1}) - \lambda_{\mathrm{STR}}(s_i)\right] + b\left(w_{\mathrm{DA}}^{\mathrm{VP}}\right)^2 N_{\mathrm{VP}} + 2\lambda_{\mathrm{bg}} \mathrm{w}_{\mathrm{bg}}^2\right) F_2,$$

where $\lambda_{\mathrm{STR}}(s_x)$ is the striatal firing rate when the agent is in state s_x. Thus, the mean membrane potential as well as the variance depend on the undiscounted difference between the values of two successive states as encoded by the striatal firing rate. If the network is not tuned to result in a constant tonic dopaminergic rate, the mean membrane potential and variance can be written in the following way:

$$\mu = \left(-\left[\gamma\lambda_{\mathrm{STR}}(s_{i+1}) - \lambda_{\mathrm{STR}}(s_i)\right] + \frac{b w_{\mathrm{DA}}^{\mathrm{VP}} N_{\mathrm{VP}}}{w_{\mathrm{DA}}^{\mathrm{STR}} N_{\mathrm{STR}}}\right) F_1$$

$$\sigma^2 = \left(-\left[\frac{w_{\mathrm{DA}}^{\mathrm{VP}}}{w_{\mathrm{DA}}^{\mathrm{STR}}}\gamma\lambda_{\mathrm{STR}}(s_{i+1}) - \lambda_{\mathrm{STR}}(s_i)\right] + \frac{b\left(w_{\mathrm{DA}}^{\mathrm{VP}}\right)^2 N_{\mathrm{VP}} + 2\lambda_{\mathrm{bg}}\mathrm{w}_{\mathrm{bg}}^2}{\left(w_{\mathrm{DA}}^{\mathrm{STR}}\right)^2 N_{\mathrm{STR}}}\right) F_2$$

with $\gamma = \left(a w_{\mathrm{DA}}^{\mathrm{VP}} N_{\mathrm{VP}}\right) / \left(w_{\mathrm{DA}}^{\mathrm{STR}} N_{\mathrm{STR}}\right)$, i.e. they depend on the discounted difference between two states. For $0 \leq \gamma \leq 1$ the phasic dopaminergic signal could therefore be used to drive temporal-difference learning in the cortico-striatal synapses on the basis of a simplified synaptic plasticity dynamics that does not compensate for a missing γ-factor (eq. 7 in Sec. 2.3 of the main text).

References

1. Ljungberg T, Apicella P, Schultz W (1992) Responses of monkey dopamine neurons during learning of behavioral reactions. J Neurophysiol 67: 145–163.

2. Burkitt AN (2006) A review on the integrate-and-fire neuron model: I. homogenous synaptic input. bicy 95: 1–19.

3. Papoulis A (1991) Probability, Random Variables, and Stochastic Processes. Boston, Massachusetts: McGraw-Hill, 3 edition.
