# Supplementary material for: An Imperfect Dopaminergic Error Signal Can Drive Temporal-Difference Learning
Source: PLoS Comput Biol. 2011 May 12;7(5):e1001133. doi: 10.1371/journal.pcbi.1001133 (PMC3093351; doi:10.1371/journal.pcbi.1001133)
Supplement: Text S2 — Mapping parameters. (0.19 MB PDF) [file pcbi.1001133.s002.pdf]

Supplementary Material S2: Mapping Parameters

1 Equivalence between the weight dynamics and the value function update

The continuous cortico-striatal synaptic plasticity dynamics

$$\dot{w_{ij}}(t) = A\Lambda_j(t)\varepsilon_j(t)\left\{(D(t) - D_{\mathrm{b}}) - G\Lambda_i(t)\right\}$$

was derived in a top-down fashion to have the same qualitative properties as the discrete-time value function update. In this section, we show that the continuous time dynamics is equivalent to the discrete-time update piecewise in Δw, where Δw is the difference between the mean cortico-striatal synaptic weights of two successive states.

We consider the mean dynamics of the synaptic weights between the population of cortical neurons representing a state s_n and the population representing the striatum:

$$\dot{w}\left(s_n, t\right) = A\lambda_{s_n}(t)\varepsilon_{s_n}(t)\left\{(\lambda_{\mathrm{d}}(t) - D_{\mathrm{b}}) - G\lambda_{\mathrm{STR}}(t)\right\} \tag{S2-1}$$

where $\lambda_{s_n}(t)$ is the mean pre-synaptic activity trace, $\varepsilon_{s_n}(t)$ the mean pre-synaptic efficacy trace, $\lambda_{\mathrm{d}}(t)$ the mean dopaminergic activity trace and $\lambda_{\mathrm{STR}}(t)$ the mean post-synaptic activity trace.

The mean of an activity trace λ_x with time constant τ can be obtained by averaging over the dynamics of the activity trace Λ_x given by Eq. (4) in the main text:

$$\dot{\lambda}_x(t) = -\frac{1}{\tau}\left(\lambda_x(t) - \lambda(t)\right)$$

where $\lambda(t)$ is the true firing rate approximated by the activity trace $\lambda_x\left(t\right)$. The solution of this inhomogeneous differential equation is

$$\lambda_x(t) = \lambda_x\left(t_0\right)e^{-(t-t_0)/\tau} - \frac{1}{\tau}\int_{t_0}^{t}\lambda\left(t'\right)e^{-\left(t-t'\right)/\tau}\,dt'$$

If $\lambda(t)$ is constant for $t > t_0$ we get:

$$\lambda_x(t) = \lambda_x(t_0)\, e^{-(t-t_0)/\tau} + \lambda\left(t_o^+\right)\left(1 - e^{-(t-t_0)/\tau}\right) . \tag{S2-2}$$

For appropriately chosen time constants of the pre-synaptic efficacy and activity traces, the plasticity of the synapse is only significant when the agent has recently exited state s_n and negligible otherwise. Assuming the transition occurs at $t = 0$, the net change in the mean synaptic weight of state s_n is :

$$\delta w(s_n) = \int_0^{\tau_{\mathrm{asp}}} \dot{w}(s_n, t)\; dt = \int_0^{\tau_{\mathrm{ph}}} \dot{w}(s_n, t)\; dt + \int_{\tau_{\mathrm{ph}}}^{\tau_{\mathrm{asp}}} \dot{w}(s_n, t)\; dt \tag{S2-3}$$

where τ_{asp} is the period for which the action neurons are suppressed so that they do not fire and τ_{ph} is the duration of the phasic activity after a state transition.

To calculate Eq. (S2-3) we need to determine expressions for $\lambda_{s_n}(t)$, $\varepsilon_{s_n}(t)$, $\lambda_{\mathrm{d}}(t)$ and $\lambda_{\mathrm{STR}}(t)$. The rate of the state neurons representing s_n is $\lambda(s)$ whilst the agent is in s_n. When the agent leaves $s_{n,}$ the neurons are no longer strongly stimulated by the environment and so the rate drops to approximately 0. Assuming a transition out of s_n at $t = 0$, the mean efficacy trace and the mean pre-synaptic activity trace are:

$$\lambda_{s_n}(t) = \lambda(s) e^{-t/\tau_{\mathrm{s}}}$$

$$\varepsilon_{s_n}(t) = 1 - e^{-t/\tau_{\varepsilon}}$$

The dopaminergic rate $\lambda_{\mathrm{d}}(t)$ is simply the constant baseline activity D_{b}, except during the phasic activity of duration τ_{ph}. The phasic firing rate λ_{ph} after a transition from a state s_n to a state s_{n+1} is a function of the weight difference of the two corresponding states. We assume this firing rate to be constant for a particular state transition. For the sake of simplicity we consider the case that the phasic activity starts at $t = 0$. From Eq. (S2-2) follows:

$$\lambda_{\mathrm{d}}(t) = \begin{cases} D_{\mathrm{b}} e^{-t/\tau_{\mathrm{d}}} & +\lambda_{\mathrm{ph}}\left(1 - e^{-t/\tau_{\mathrm{d}}}\right) \text{ for } t \in [0, \tau_{\mathrm{ph}}] \\ \lambda_{\mathrm{ph}} e^{-(t-\tau_{\mathrm{ph}})/\tau_{\mathrm{d}}} & +D_{\mathrm{b}}\left(1 - e^{-(t-\tau_{\mathrm{ph}})/\tau_{\mathrm{d}}}\right) \text{ for } t \in [\tau_{\mathrm{ph}}, \tau_{\mathrm{asp}}] \end{cases}$$

The post-synaptic activity $\lambda_{\mathrm{STR}}(t)$ depends on the input from the currently active state, i.e. s_n whilst the agent is in s_n, and s_{n+1} after the transition at $t = 0$. Therefore, for $t \in [0, \tau_{\mathrm{asp}}]$ the mean post-synaptic activity trace is given by

$$\lambda_{\mathrm{STR}}(\mathrm{t}) = \lambda_{\mathrm{STR}}(s_n)\, e^{-t/\tau_{\mathrm{STR}}} + \lambda_{\mathrm{STR}}(s_{n+1})\left(1 - e^{-t/\tau_{\mathrm{STR}}}\right)$$

The mean synaptic weight change is:

$$\delta w\left(s_n\right) = A\lambda(s)\left\{\lambda_{\mathrm{ph}}T_1 - C\left(\lambda_{\mathrm{STR}}\left(s_n\right)T_2 + \lambda_{\mathrm{STR}}\left(s_{n+1}\right)T_3\right) + D_{\mathrm{b}}T_4\right\} \tag{S2-4}$$

with

$$\begin{aligned}
T_1 &= \left(\hat{d}\left(\frac{1}{\tau_{\mathrm{s}}}+\frac{1}{\tau_{\mathrm{d}}}\right) - \hat{d}\left(\frac{1}{\tau_{\mathrm{s}}}+\frac{1}{\tau_{\mathrm{d}}}+\frac{1}{\tau_{\varepsilon}}\right)\right)e^{\tau_{\mathrm{ph}}/\tau_{\mathrm{d}}} + \hat{\tau}\left(\frac{1}{\tau_{\mathrm{s}}}\right) - \hat{\tau}\left(\frac{1}{\tau_{\mathrm{s}}}+\frac{1}{\tau_{\varepsilon}}\right) - \hat{\tau}\left(\frac{1}{\tau_{\mathrm{s}}}+\frac{1}{\tau_{\mathrm{d}}}\right) + \hat{\tau}\left(\frac{1}{\tau_{\mathrm{s}}}+\frac{1}{\tau_{\mathrm{d}}}+\frac{1}{\tau_{\varepsilon}}\right)\\
T_2 &= \hat{k}\left(\frac{1}{\tau_{\mathrm{s}}}+\frac{1}{\tau_{\mathrm{STR}}}\right) - \hat{k}\left(\frac{1}{\tau_{s}}+\frac{1}{\tau_{\varepsilon}}+\frac{1}{\tau_{\mathrm{STR}}}\right)\\
T_3 &= \hat{k}\left(\frac{1}{\tau_{\mathrm{s}}}\right) - \hat{k}\left(\frac{1}{\tau_{\mathrm{s}}}+\frac{1}{\tau_{\varepsilon}}\right) - \hat{k}\left(\frac{1}{\tau_{\mathrm{s}}}+\frac{1}{\tau_{\mathrm{STR}}}\right) + \hat{k}\left(\frac{1}{\tau_{\mathrm{s}}}+\frac{1}{\tau_{\mathrm{STR}}}+\frac{1}{\tau_{\varepsilon}}\right)\\
T_4 &= \hat{\tau}\left(\frac{1}{\tau_{\mathrm{s}}}+\frac{1}{\tau_{\mathrm{d}}}\right) - \hat{\tau}\left(\frac{1}{\tau_{\mathrm{s}}}+\frac{1}{\tau_{\mathrm{d}}}+\frac{1}{\tau_{\varepsilon}}\right) + \hat{d}\left(\frac{1}{\tau_{\mathrm{s}}}\right) - \hat{d}\left(\frac{1}{\tau_{\mathrm{s}}}+\frac{1}{\tau_{\varepsilon}}\right) - \hat{d}\left(\frac{1}{\tau_{\mathrm{s}}}+\frac{1}{\tau_{\mathrm{d}}}\right)e^{\tau_{\mathrm{ph}}/\tau_{\mathrm{d}}}\\
&\quad + \hat{d}\left(\frac{1}{\tau_{\mathrm{s}}}+\frac{1}{\tau_{\mathrm{d}}}+\frac{1}{\tau_{\varepsilon}}\right)e^{\tau_{\mathrm{ph}}/\tau_{\mathrm{d}}} - \hat{k}\left(\frac{1}{\tau_{\mathrm{s}}}\right) + \hat{k}\left(\frac{1}{\tau_{\mathrm{s}}}+\frac{1}{\tau_{\varepsilon}}\right)
\end{aligned}$$

and

$$\begin{aligned}
\hat{\tau}(x) &= 1/x\left(1 - e^{-x\tau_{\mathrm{ph}}}\right)\\
\hat{d}(x) &= 1/x\left(e^{-x\tau_{\mathrm{ph}}} - e^{-x\tau_{\mathrm{asp}}}\right)\\
\hat{k}(x) &= 1/x\left(1 - e^{-x\tau_{\mathrm{asp}}}\right)\ .
\end{aligned}$$

One major difference between the traditional TD error and the dopaminergic signal is that the dopaminergic firing rate λ_{ph} depends non-linearly on successive reward estimates, Δw, whereas the TD error is a linear function of successive value functions (see Fig. 4 in the main text). However, is is possible to approximate the non-linear function for a given reward signal piecewise in Δw by a linear function:

$$\lambda_{\mathrm{ph}} = m_{\mathrm{d}}\Delta w + c_{\mathrm{d}} \tag{S2-5}$$

To compare the synaptic weight change to the value function update we map the value function to the units of synaptic weights:

$$V(s) = m_V\lambda_{\mathrm{STR}}(s) + c_V \tag{S2-6}$$

$$\lambda_{\mathrm{STR}}(s) = m_\lambda w(s) + c_\lambda\ . \tag{S2-7}$$

The linear relationship Eq. (S2-7) is fulfilled for $w \in [30, 100]\,\mathrm{pA}$ with $m_\lambda = 0.43\frac{\mathrm{Hz}}{\mathrm{pA}}$ and $c_\lambda = -3.93\,\mathrm{Hz}$. Within a given range of Δw the synaptic weight change Eq. (S2-4) can be written with Eq. (S2-6) and Eq. (S2-7) as:

Δw [pA]	I_{r}[pA]	m_{d} [Hz·pA^{-1}]	c_{d} [Hz]
> 10	0	18.72	-52.92
$[-20, 10]$	0	5.6	120.97
$[-60, -20[$	0	0.47	29.5
≥ -10	600	27.4	967.08
< -10	600	13.34	831.27

Table S2-1: The dependence of the linear coefficients m_{d} and c_{d} of equation Eq. (S2-5) on $\Delta w = w(s_{n+1}) - w(s_n)$ and the reward amplitude I_{r} for the parameters chosen in our simulation.

$$\delta w(s_n) = \frac{1}{m_\lambda m_V} \delta V(s_n)$$

with

$$\delta V(s_n) = \alpha(\gamma V(s_{n+1}) - V(s_n) + \kappa)$$

and

$$\begin{aligned}
\alpha = \alpha(m_{\mathrm{d}}) &= m_\lambda A \lambda(s)(m_{\mathrm{d}} T_1 / m_\lambda + G T_2) \\
\gamma = \gamma(m_{\mathrm{d}}) &= \frac{m_{\mathrm{d}} T_1 / m_\lambda - G T_3}{m_{\mathrm{d}} T_1 / m_\lambda + G T_2} \\
\kappa = \kappa(m_{\mathrm{d}}, c_{\mathrm{d}}) &= \frac{c_{\mathrm{d}} T_1 + G c_V (T2 + T3)/m_V + D_{\mathrm{b}} T_4}{m_{\mathrm{d}} T_1 / (m_\lambda m_V) + G T_2 / m_V}
\end{aligned} \tag{S2-8}$$

Because m_{d} and c_{d} are dependent on the range of Δw and the direct current applied to the dopamine neurons I_{r}, the weight update δw can be interpreted as a TD(0) learning value function update with self-adapting learning parameters and a self-adapting offset that depend on the current weight change and reward.

The values of m_{d} and c_{d} for the parameters chosen in our simulations for a direct current of $I_{\mathrm{r}} = 600\,\mathrm{pA}$ and $I_{\mathrm{r}} = 0\,\mathrm{pA}$ are summarized in Table S2-1.

2 Policy mapping

The probability of choosing a certain action in a certain state is given by the probability that the actor neuron encoding the action fires first in response to the input from the cortical neurons representing the state. This probability depends on the mean strength of the synapses connecting the cortical 'state' neurons to the actor neuron in comparison to the mean strength of the synapses connecting the state neurons to the other competing actor neurons. A mapping of synaptic weights to probabilities for a similar architecture was derived in [1]. To obtain the mapping, first spike time distributions are measured as a function of synaptic weight and fitted with a gamma probability densitiy function $f(t|\kappa, \theta) = \frac{1}{\theta^\kappa \Gamma(\kappa)} t^{\kappa-1} e^{-\frac{t}{\theta}}$, where Γ is the gamma function. The probability that an actor neuron p with mean synaptic weights w_p fires before an actor neuron

q with mean synaptic weights w_q is given by:

$$P\left(t_p^{\mathrm{fs}} < t_q^{\mathrm{fs}} | w_p, w_q\right) = \int_0^{\infty} f\left(t | \kappa\left(w_p\right), \theta\left(w_p\right)\right) \left[1 - \gamma\left(\frac{t}{\theta\left(w_q\right)}, \kappa\left(w_q\right)\right)\right] dt.$$

Here, $\gamma(t, \kappa)$ is the incomplete gamma function, with $\gamma(t, \kappa) = \frac{1}{\Gamma(\kappa)} \int_0^t e^{-t} t^{\kappa-1} dt$.

The policy defined by the probabilities of the respective action neurons firing first is not identical to the Gibbs softmax method used to select actions in the discrete-time algorithmic implementation (see introduction). However, the precise non-linear function used to select actions is not critical; any selection mechanism that predominantly selects the most preferred action but occasionally selects a less preferred action would be expected to generate a similar policy.

3 Discrete-time simulation

In order to have a reference for the learning performance of the neuronal network in the grid-world task, we implemented in C++ a classical algorithmic discrete-time actor-critic TD(0) learning agent as described in the introduction. We mapped the synaptic parameters to the discrete-time learning parameters such that they result in the same value function update in the range $\Delta w \in [-20, 10]\,\mathrm{pA}$ (see Sec. 1 for a derivation of this relationship). For the synaptic parameters used in our study, this leads to learning parameters $\alpha = 0.4$ and $\gamma = 0.9$. We adapted the algorithm such that a value function update is only carried out if the chosen action leads to a state transition, in line with the neuronal dynamics. However, removing this constraint does not result in an increased performance for the discrete-time algorithm (data not shown).

For certain ranges of Δw the reward defined for the neuronal system, i.e. the direct current applied to the dopamine neurons I_{r}, can also be mapped to the real-valued reward defined in the discrete-time TD(0) algorithm (see Sec. 1). This mapping depends on the parameters m_V and c_V, which map the value function V to firing rates according to Eq. (S2-6). These parameters have no neuronal correlates, so we determined the reward by performing a parameter scan over possible values for m_V and c_V. For each parameter set we calculated the discrete-time reward according to the derived mapping for $\Delta w < -10\,\mathrm{pA}$. We then let the discrete-time algorithmic TD(0) learning agent solve the grid-world task for that reward and measured the resultant minimum and maximum equilibrium value functions across all states, $V_{\mathrm{min}} = \min_{\mathrm{s}} V(\mathrm{s})$ and $V_{\mathrm{max}} = \max_{\mathrm{s}} V(\mathrm{s})$. The minimum and maximum values of the value function for the neuronal implementation are given by $V\left(w_{\mathrm{min}}\right)$ and $V\left(w_{\mathrm{max}}\right)$, where $V(w) = m_V m_\lambda w + m_V c_\lambda + c_V$ (see Eq. (S2-6) and Eq. (S2-5)) and $w_{\mathrm{min}} = \min_{\mathrm{s}} w(\mathrm{s})$ and $w_{\mathrm{max}} = \max_{\mathrm{s}} w(\mathrm{s})$ are the minimum and maximum of $w(\mathrm{s})$ over all states; where $w(\mathrm{s})$ is the mean equilibrium cortico-striatal synaptic weight belonging to a state s. We assume that the rewards applied to the neuronal and algorithmic models are equivalent if the minimum and maximum values of the equilibrium value function are equivalent. We therefore select values of m_V and c_V that minimize the function $|V_{\mathrm{min}} - V\left(w_{\mathrm{min}}\right)| + |V_{\mathrm{max}} - V\left(w_{\mathrm{max}}\right)|$, resulting in $m_V = 0.6\,\mathrm{s}$, $c_V = -10.0$ and a reward for the discrete-time TD(0) implementation of $r = 12.161$.

The discrete-time algorithmic implementation selects actions by the Gibbs softmax method (see introduction). As this

nonlinear function cannot be mapped to the neuronal action selection process on the basis of first spike time probabilities, we arbitrarily set the learning parameter for the policy update to $\beta = 0.3$. The learning behaviour is not particularly sensitive to the choice of β in the range $[0.1, 0.5]$; for higher values the learning is less stable leading to a worse equilibrium performance (data not shown). Similarly to the neuronal implementation, we restrict the maximal and minimal probabilities of selecting an action by restricting the maximal and minimal values for the action preferences p to the range $[1, 5.8]$. This results in a maximum probability of choosing an action of 97.59%, as for the neuronal implementation, and a minimal probability of 0.27%, compared to the value of 2.82% in the neuronal implementation. If this contraint is relaxed, the discrete-time algorithmic implementation results in a slightly better equilibrium performance.

References

1. Potjans W, Morrison A, Diesmann M (2009) A spiking neural network model of an actor-critic learning agent. Neural Comput 21: 301–339.
